# A Prenylated dsRNA Sensor Protects Against Severe COVID-19

**DOI:** 10.1126/science.abj3624

**Published:** 2021-10-29

**Authors:** Arthur Wickenhagen, Elena Sugrue, Spyros Lytras, Srikeerthana Kuchi, Marko Noerenberg, Matthew L. Turnbull, Colin Loney, Vanessa Herder, Jay Allan, Innes Jarmson, Natalia Cameron-Ruiz, Margus Varjak, Rute M. Pinto, Jeffrey Y Lee, Louisa Iselin, Natasha Palmalux, Douglas G. Stewart, Simon Swingler, Edward J.D. Greenwood, Thomas W.M. Crozier, Quan Gu, Emma L. Davies, Sara Clohisey, Bo Wang, Fabio Trindade Maranhão Costa, Monique Freire Santana, Luiz Carlos de Lima Ferreira, Lee Murphy, Angie Fawkes, Alison Meynert, Graeme Grimes, ISARIC4C investigators, Joao Luiz Da Silva Filho, Matthias Marti, Joseph Hughes, Richard J. Stanton, Eddie C.Y. Wang, Antonia Ho, Ilan Davis, Ruth F. Jarrett, Alfredo Castello, David L. Robertson, Malcolm G. Semple, Peter J.M. Openshaw, Massimo Palmarini, Paul J. Lehner, J. Kenneth Baillie, Suzannah J. Rihn, Sam J. Wilson

**Affiliations:** 1 MRC−University of Glasgow Centre for Virus Research (CVR), Institute of Infection, Inflammation and Immunity, University of Glasgow; Glasgow, UK; 2 Department of Biochemistry, University of Oxford, South Parks Road, Oxford, UK; 3 Nuffield Department of Medicine, Peter Medawar Building for Pathogen Research, University of Oxford, Oxford, UK; 4 Cambridge Institute of Therapeutic Immunology and Infectious Disease, Jeffrey Cheah Biomedical Centre, Cambridge Biomedical Campus, University of Cambridge; Cambridge, UK; 5 Roslin Institute, University of Edinburgh; Edinburgh, UK; 6 laboratory of Tropical Diseases − Prof. Luiz Jacintho da Silva, Department of Genetics, Evolution, Microbiology and Immunology, Institute of Biology, University of Campinas; Campinas, SP, Brazil; 7 Department of Education and Research, Oncology Control Centre of Amazonas State − 5 FCECON; Manaus, AM, Brazil; 8 Postgraduate Program in Tropical Medicine, Tropical Medicine Foundation Dr. Heitor Vieira Dourado, Manaus, AM, Brazil; 9 Edinburgh Clinical Research Facility, University of Edinburgh, Western General Hospital, Edinburgh, UK; 10 MRC Human Genetics Unit, Institute of Genetics and Molecular Medicine, University of Edinburgh, Western General Hospital, Edinburgh, UK; 12 Wellcome Centre for Molecular Parasitology, Institute of Infection, Immunity and Inflammation, University of Glasgow; Glasgow, UK; 13 Division of Infection & Immunity, Cardiff University; Cardiff, UK; 14 NIHR Health Protection Research Unit for Emerging and Zoonotic Infections, Institute of Infection, Veterinary and Ecological Sciences, University of Liverpool, Liverpool, UK; 15 Respiratory Medicine, Alder Hey Children’s Hospital, Liverpool, UK; 16 National Heart and Lung Institute, Imperial College London, London, UK; 17 Imperial College Healthcare NHS Trust London, London, UK; 18 Intensive Care Unit, Royal Infirmary of Edinburgh, Edinburgh, UK

## Abstract

Inherited genetic factors can influence the severity of COVID-19, but the molecular explanation underpinning a genetic association is often unclear. Intracellular antiviral defenses can inhibit the replication of viruses and reduce disease severity. To better understand the antiviral defenses relevant to COVID-19, we used interferon-stimulated gene (ISG) expression screening to reveal that OAS1, through RNase L, potently inhibits SARS-CoV-2. We show that a common splice-acceptor SNP (Rs10774671) governs whether people express prenylated OAS1 isoforms that are membrane-associated and sense specific regions of SARS-CoV-2 RNAs, or only express cytosolic, nonprenylated OAS1 that does not efficiently detect SARS-CoV-2. Importantly, in hospitalized patients, expression of prenylated OAS1 was associated with protection from severe COVID-19, suggesting this antiviral defense is a major component of a protective antiviral response.

Severe acute respiratory syndrome coronavirus 2 (SARS-CoV-2), the virus responsible for the COVID-19 pandemic, first emerged in humans in 2019 and has left an indelible mark on global health, culture and prosperity. SARS-CoV-2 is particularly sensitive to inhibition by type I interferons (IFNs), and because type I IFNs heavily influence COVID-19 outcome, there is great interest in understanding how individual IFN-stimulated genes (ISGs) inhibit SARS-CoV-2. Specifically, allelic variants of genes within the IFN system are associated with severity of COVID-19 (1). Moreover, neutralizing anti-IFN autoantibodies likely prevent host IFN responses from controlling SARS-CoV-2 replication (2), promoting severe COVID-19. Accordingly, recombinant IFNs have therapeutic potential (3) although the correct timing of IFN responses or the administration of recombinant IFNs is likely critical (4).

## ISG expression screening identifies candidate anti-SARS-CoV-2 effectors

We hypothesized that variation in individual ISGs likely underlies some of the observed differential susceptibility to severe COVID-19. To identify the ISG products that inhibit SARS-CoV-2, we used arrayed ISG expression screening (5, 6). We first confirmed that SARS-CoV-2 was IFN-sensitive in transformed human lung A549 cells that were modified to express the SARS-CoV-2 receptor, ACE2, and the serine protease, TMPRSS2 (7, 8). While these cells supported efficient viral replication, SARS-CoV-2 was potently inhibited by type I IFN treatment in this context (fig. S1A,B). Thus, A549 cells were deemed suitable for ISG expression screening.

**Figure 1:**
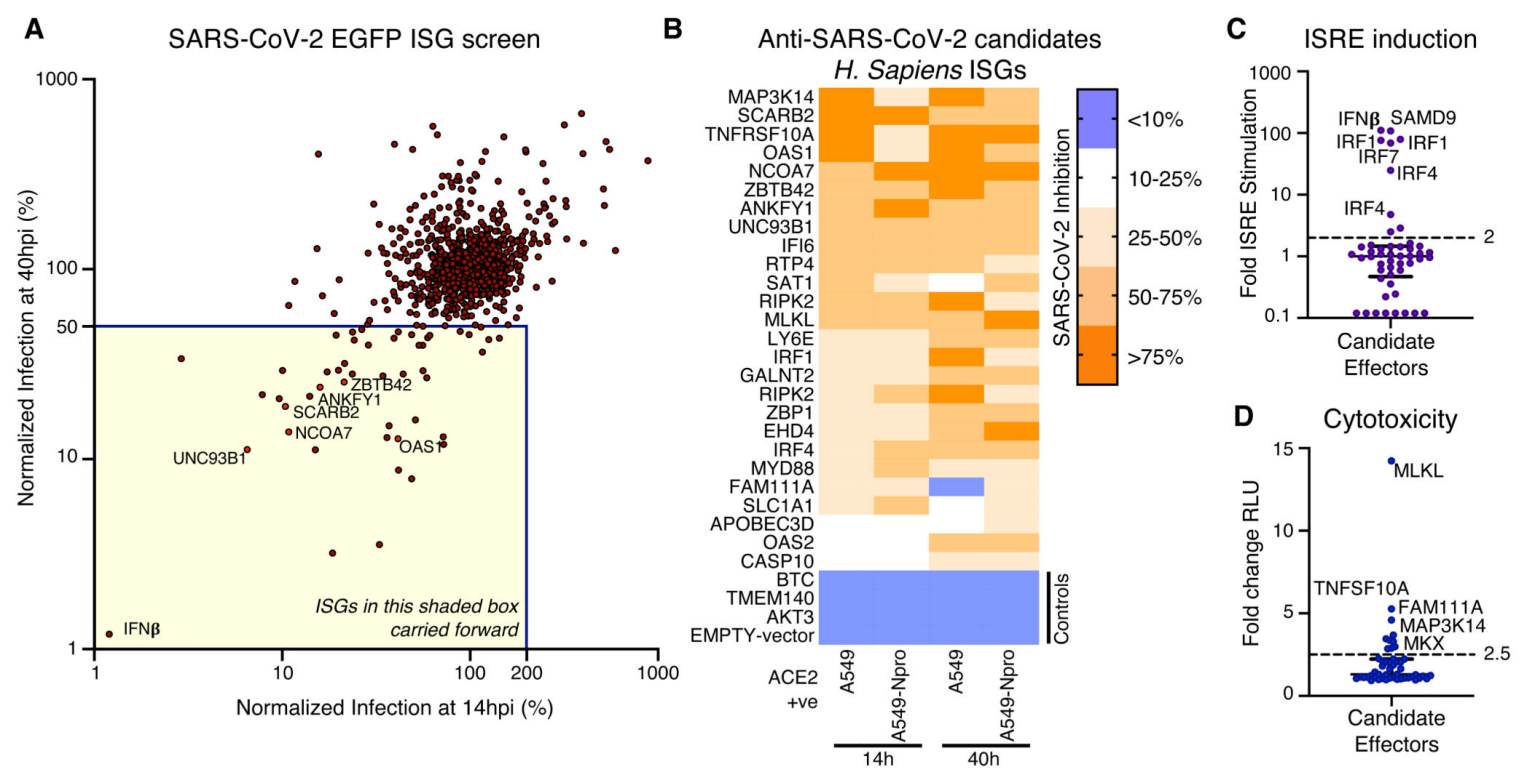
Arrayed ISG expression screening reveals factors with candidate anti-SARS-CoV-2 activity. (A) A549-Npro-ACE2 cells were transduced with hundreds of individual human or macaque ISGs (see Supplementary Figure 1C-E) and infected with SARS-CoV-2-EGFP (Wuhan-1), in duplicate, and the level of infection in the presence of each ISG was measured using flow cytometry at 14 and 40 hpi. (B) Miniscreen of the ability of human candidate effectors identified in (A) alongside controls, to inhibit SARS-CoV-2 in A549 and A549-Npro at 14 and 40 hpi (the equivalent panel for macaque ISGs presented in Supplementary Figure 1L). (C,D) The ability of each human and macaque effector to either stimulate ISRE activity using A549-ISRE-EGFP cells (C) or cause toxicity (CytoTox-Glo) using supernatant from the same A549-ISRE-EGFP cells (D) at 48 hours post-transduction with the relevant ISG-encoding lentiviral vector. The dashed line indicates threshold for negative selection.

Since exogenously expressed ISGs can trigger antiviral gene expression pathways (5), we used transformed ACE2-expressing and IRF3-deficient A549 cells (A549-Npro-ACE2), which possess an attenuated ability to produce IFN (9) as the background for the screens. We transduced these cells with an arrayed library of lentiviral vector-encoded ISGs in a 96-well plate format (one ISG per well), using a library of >500 human ISGs and a similar library of >350 rhesus macaque ISGs (5) (fig. S1C-E). The macaque ISGs were included as they increased the total number of ISGs under consideration (including orthologs and additional ISGs). Importantly, around two-thirds of the ISGs examined could potentially be relevant to betacoronavirus infection (10) (fig. S1F,G). To capture inhibition that might occur at different stages of the virus lifecycle, we used a GFP-encoding recombinant SARS-CoV-2 (11) and measured the ability of each individual ISG to inhibit SARS-CoV-2 at 14 hours (early) and 40 hours (late) post-infection. Using this approach, we identified several candidate anti-SARS-CoV-2 effectors (Fig. 1A). All ISGs that conferred >2-fold inhibition at early and late timepoints, or only at late timepoints, were considered potential ‘candidates’ and underwent further independent confirmatory ‘miniscreens’. The magnitude of protection conferred by each candidate ISG at early and late timepoints was assessed using ACE2-positive cells in the presence or absence of IRF3 (Fig. 1B and fig. S1H-L). In addition, we sought to subtract nonspecific inhibitors of SARS-CoV-2 by identifying ISGs that triggered a polygenic ‘antiviral state’ (Fig. 1C) or caused cytotoxicity (Fig. 1D). Following these confirmatory and negative selection screens, we identified six candidate antiviral effectors that robustly inhibited SARS-CoV-2 (without inducing substantial toxicity or inducing ISRE expression).

These candidate effectors included known antiviral genes such as the IFN-inducible short isoform (isoform 4) of NCOA7, which inhibits influenza A viruses (IAVs) (12) and OAS1, a dsRNA sensor capable of activating RNase L (13, 14). We also identified UNC93B1, a polytopic membrane protein required for TLR trafficking (15) as well as SCARB2, a virus receptor (16) involved in cholesterol transport (17, 18). In addition, we identified ANKFY1 and ZBTB42, which have not previously been ascribed antiviral activity. We exogenously expressed these ISGs in human A549 lung cells modified to express either ACE2, or both TMPRSS2 and ACE2, and examined their ability to inhibit an isolate of SARS-CoV-2 (CVR-GLA-1 isolated in March 2020 (8)).

**Figure 2:**
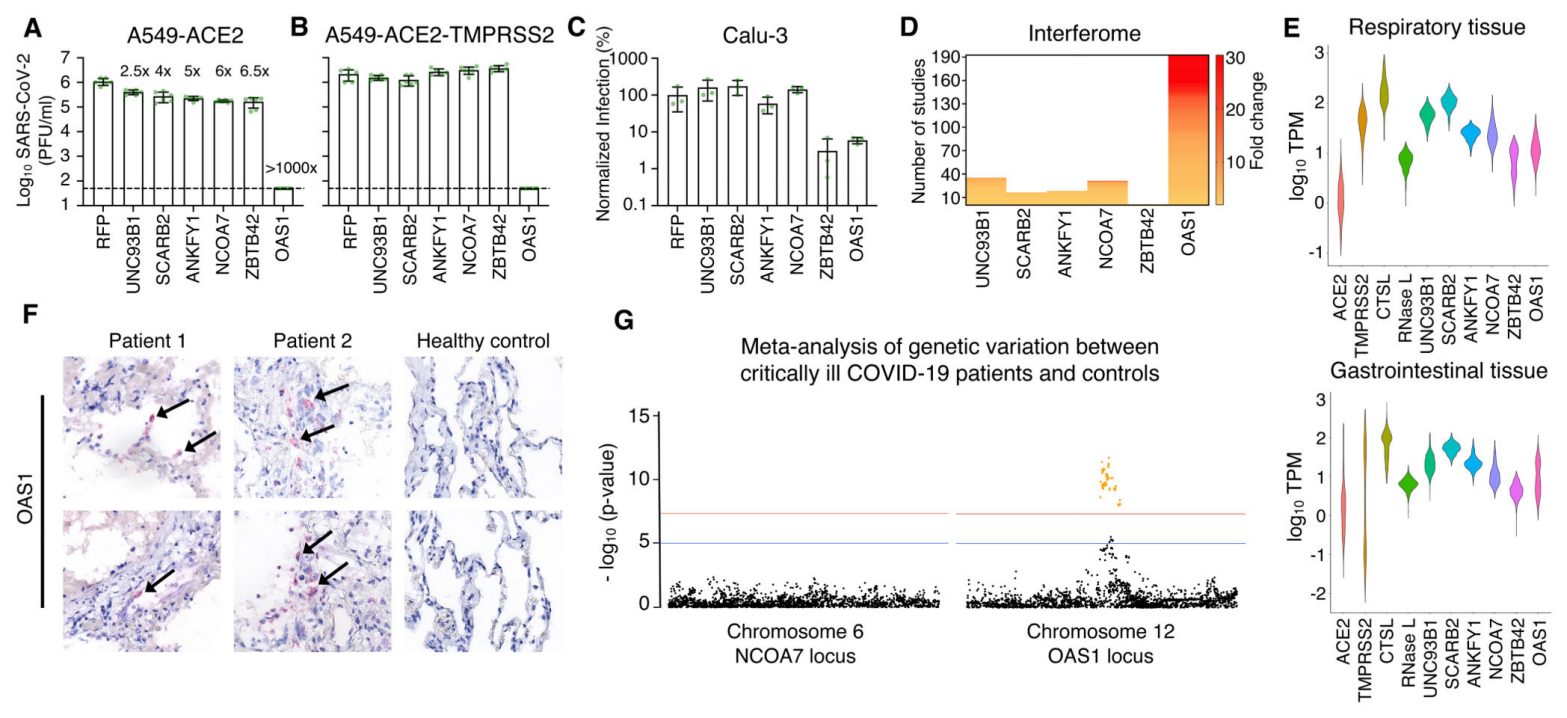
The ISG OAS1 initiates a block to SARS-CoV-2 replication. (A-B) SARS-CoV-2 isolate CVR-GLA-1 infectious titers (PFU/ml) were determined using A549-ACE2 (A) or A549-ACE2-TMPRSS2 cells (B) modified to express the candidate effectors (UNC93B1, SCARB2, ANKFY1, NCOA7, ZBTB42 or OAS1) from the screening pipeline (Fig. 1 A-D). Fold protection from SARS-CoV-2 is indicated for each gene in (A). (C) SARS-CoV-2-ZsGreen infectious titers on Calu-3 cells expressing the same hit ISGs as in (A,B), measured by flow cytometry at 40 hpi. (D) ‘ISG-ness’ of selected genes was assessed by fold change upon type I IFN stimulation as reported in studies in the Interferome v2.01 database (interferome.org). (E) Gene expression analysis across different respiratory and gastrointestinal tissues using datasets from the Genotype-Tissue Expression (GTEx) database, with ACE2 and TMPRSS2 included for reference. RNase L included as functionally linked to OAS1. (F) Detection of OAS1 gene expression by RNAscope in FFPE lung tissue of deceased COVID-19 patients compared to healthy control lung tissue. Arrows indicate staining +ve cells. (G) Meta-analysis of the COVID-19 Host Genetics Initiative (covid19hg.org) for genetic variation between critical ill COVID-19 patients and control populations at the gene locus of the NCOA7 and OAS1 genes with the red line indicating the threshold for significant SNPs (yellow dots).

Interestingly, in the absence of TMPRSS2, all six candidate antiviral effectors inhibited SARS-CoV-2 (2.5-fold- >1000-fold) (Fig. 2A), whereas only OAS1 consistently inhibited this ‘early’ isolate in different cell backgrounds, irrespective of TMPRSS2 status (Fig. 2A-C). Indeed, TMPRSS2-mediated entry has been proposed as a strategy used by SARS-CoV-2 to evade antiviral factors (19), including NCOA7 (20). Moreover, the initial screens were executed in the absence of TMPRSS2 (Fig. 1) potentially biasing our ‘hit list’ towards effectors that might be inactive in TMPRSS2-expressing cells.

## OAS1 exhibits multiple characteristics expected from an anti-SARS-CoV-2 effector

To identify effectors present at the sites of SARS-CoV-2 infection, we examined the interferon responsivity of the 6 candidate effectors using published studies from the interferome v2.0 database (Fig. 2D) (21). We also determined their basal expression in respiratory and gastrointestinal tracts (GTEx) (Fig. 2E) and assessed their transcript abundance in post-mortem lung tissue from COVID-19 patients (Fig. 2F and fig. S2A). In addition, we examined the genomic locus of each candidate effector for single nucleotide polymorphisms (SNPs) within alleles that may be associated with increased susceptibility to infection and/or severe disease (Fig. 2G and fig. S2B). In accordance with a potential role in influencing susceptibility to SARS-CoV-2 infection (22), OAS1 was frequently detected in nasal epithelium (which is a potential site of initial infection (23)) sampled from healthy individuals (3/6 individuals) (fig. S2C). Following these analyses, we focused our attention on OAS1 as the antiviral activity was the most robust (Fig. 2A-C), the low basal transcription was highly IFN inducible (Fig. 2D,E), the mRNA was readily detectable in infected patients (Fig. 2F) and common allelic variants were associated with altered susceptibility to infection and severe disease (Fig. 2G) (1, 22, 24, 25).

## The block initiated by OAS1 is not dependent on OAS3

**Figure 3:**
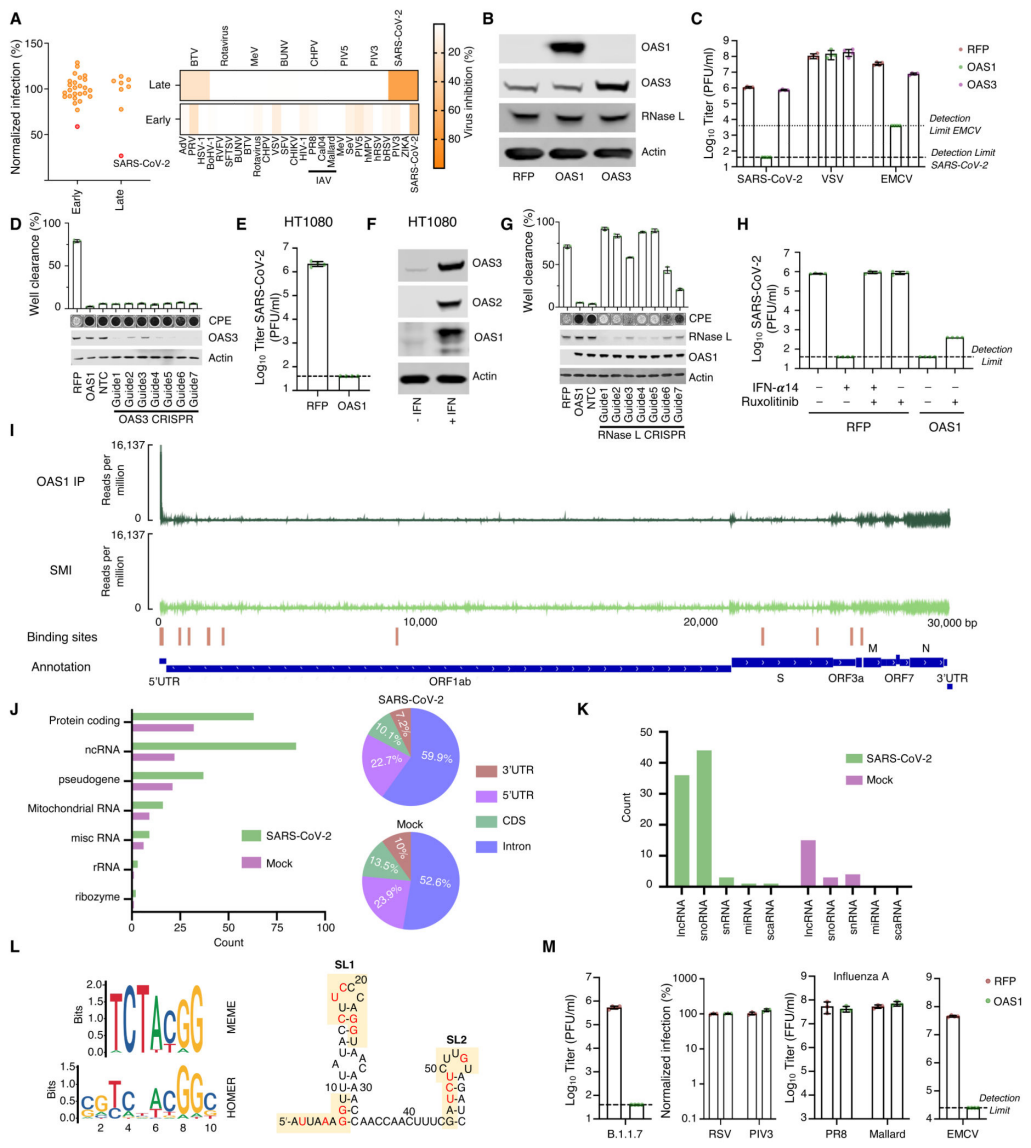
OAS1 inhibition of SARS-CoV-2 is specific and mediated via the RNase L pathway. (A) Normalized infection in the presence of OAS1 at early or late stages of the viral life cycle, quantified in large-scale ISG expression screens (similar to Figure 1) for a panel of viruses (described in arrayed ISG expression screening methods). (B) A549-ACE2-TMPRSS2 (AAT) cells were modified to express OAS1 and OAS3 and protein expression (OAS1, OAS3 and RNase L) in the cell lines was monitored using western blotting. (C) The titers of SARS-CoV-2, VSV and EMCV were determined (PFU/ml) in the presence of each ISG in the cell lines characterized in B. (D) SARS-CoV-2 replication (well clearance at 72 hpi due to cytopathic effects of virus replication) was assessed in AAT cells that were modified to express exogenous OAS1, and whose OAS3 expression was reduced using seven different lentiviral vector-derived CRISPR guides and one non-targeting control guide (NTC). The level of OAS3 KO was assessed by western blotting and typical virus induced CPE is shown. (E) SARS-CoV-2 infectious titer (PFU/ml) on HT1080-ACE2-TMPRSS2 (HAT) cells expressing RFP or OAS1. (F) Protein expression (OAS3, OAS2 and OAS1) in HAT cells, in the presence and absence of pretreatment with 1000 pg/ml IFN***α***14, monitored by western blotting. (G) SARS-CoV-2 replication (well clearance at 72 hpi due to cytopathic effects of virus replication) was assessed in cells whose RNase L expression was reduced using seven different lentiviral vector-derived CRIPSR guides and one non-targeting control guide (NTC). The level of RNase L KO was assessed by western blotting and typical virus induced CPE is shown. (H) SARS-CoV-2 infectious titer (PFU/ml) on AAT cells expressing RFP or OAS1 was determined in the presence and absence of pretreatment with 100 U/ml IFN***α***14 and/or 0.5 μM Ruxolitinib. (I) iCLIP2 analysis of OAS1 binding sites on SARS-CoV-2 RNA. Coverage of 3 replicate tracks overlaid mapped to the SARS-CoV-2 genome in the OAS1-IP and a size matched input control (SMI) allow detection of OAS1 binding sites shown in red above the SARS-CoV-2 genome annotation. (J) Transcriptome-wide profiling of OAS1 iCLIP2 targets by gene biotypes in SARS-CoV-2 or mock infected cells. Pie charts indicate distribution of OAS1 binding sites within each transcript feature of protein coding genes. (K) Detailed representation of OAS1 iCLIP2 targets in non-coding RNA biotypes between SARS-CoV-2 and mock infected samples. (L) Motif prediction of OAS1 binding sites in cellular transcripts using MEME or HOMER. Presence of these predicted binding motifs in the SL1 and SL2 loops of the 5’-UTR of SARS-CoV-2 (37). (M) AAT cells were modified to express RFP or OAS1. The infectious titer of SARS-CoV-2 B.1.1.7 and EMCV on these cells was determined by plaque assay. Similarly, titers of RSV-GFP and PIV3-GFP were determined using flow cytometry (24 hpi). Titers of influenza A viruses (IAV/H1N1/PR8 and IAV/H1N1/Mallard) were determined using an immunostained focus forming assay.

OAS1 is an evolutionarily ancient ISG that has maintained IFN responsivity for hundreds of millions of years (26). The OAS system was one of the first antiviral pathways to be defined (27) and the canonical model of OAS antiviral activity involves initial dsRNA sensing by OASs, which results in the synthesis of 2′-5′-linked oligoadenylates (2-5A). 2-5A induces the dimerization of inactive RNase L, which then mediates the indiscriminate cleavage of viral and host RNAs presenting single-stranded UpU and UpA motifs (28). The initial sensing of virus dsRNA that subsequently activates RNase L has mostly been ascribed to OAS3, with OAS1 infrequently considered as a major viral dsRNA sensor (29). Indeed, in over 30 arrayed ISG screens completed in our laboratory, SARS-CoV-2 was the only virus substantially inhibited by OAS1 (Fig. 3A). We therefore investigated whether OAS1 antiviral activity was dependent upon OAS3. OAS3 is readily detectable in A549 cells (Fig. 3B) and additional exogenous OAS3 had no effect on the replication of SARS-CoV-2 (Fig. 3B,C). Importantly, removal of OAS3 to undetectable levels using CRISPR-Cas9 did not attenuate the ability of exogenous OAS1 to instigate a block to SARS-CoV-2 (Fig. 3D). Similarly, exogenous expression of OAS1 initiated a block to SARS-CoV-2 in HT1080 cells, which have low or undetectable levels of basal OAS expression (Fig. 3E,F). Thus, OAS3 is not required for OAS1 to instigate a block to SARS-CoV-2 replication. To confirm that OAS1 was inhibiting SARS-CoV-2 through the synthesis of 2-5A and activated RNase L, we next disrupted the RNase L locus in OAS1-expressing cells. The antiviral activity of OAS1 was only effective in the presence of RNase L and the loss of RNase L abrogated the ability of OAS1 to inhibit SARS-CoV-2 (Fig. 3G). RNase L activation can, in principle, inhibit viruses by degrading viral or host RNAs (30, 31), eventually resulting in apoptosis (32), or by triggering an IFN response (33). We therefore examined the contribution of RNase L-induced IFN responses to the inhibition of SARS-CoV-2 by OAS1 by ablating JAK-STAT signaling using the Janus kinase (JAK) inhibitor ruxolitinib (Rux) (34). Type I IFN treatment potently inhibited SARS-CoV-2 replication (Fig. 3H), and this effect was entirely reversed by the addition of Rux. Importantly, OAS1 potently inhibited SARS-CoV-2 in the absence of JAK-STAT signaling (Fig. 3H), indicating that the RNase L-mediated destruction of host and/or viral RNAs is likely the predominant mechanism through which OAS1 inhibits SARS-CoV-2.

## OAS1 senses conserved dsRNA structures in the SARS-CoV-2 5′-UTR

To understand how OAS1 senses SARS-CoV-2 infection, we applied individual nucleotide-resolution crosslinking and immunoprecipitation (iCLIP2) (35) to SARS-CoV-2 infected AAT cells. To maximise the viral RNA (vRNA) available to OAS1, we did this in AAT cells modified to express exogenous OAS1, that were also devoid of substantial RNase L activity (guide 5, Fig. 3G). The iCLIP approach freezes protein-RNA interactions using ultraviolet crosslinking, followed by RNase trimming to generate protein-protected RNA fragments. OAS1 can subsequently be immunoprecipitated and the crosslinked RNA reverse transcribed and sequenced. Because the amino acids crosslinked to the RNA cause termination of reverse transcription, iCLIP2 provides a single-nucleotide resolution map of protein binding sites within RNA molecules. We used control IgG immunoprecipitation and size-matched input controls (36) to subtract confounding sequences not derived from OAS1 binding. Strikingly, iCLIP2 revealed that OAS1 interacted with several regions of the SARS-CoV-2 genome (table S1), with the most prominent sites mapping to the first 54 nucleotides of the 5′ untranslated region (UTR) (37), present in all SARS-CoV-2 positive-sense viral RNAs (Fig. 3I). No significant traces of binding were observed in the negative strand, suggesting that OAS1 likely bound positive-sense viral transcripts (as opposed to replication intermediates). The major viral target encompassed stem loops 1 and 2 (SL1 and SL2) within the 5′-UTR, consistent with the known capacity of OAS1 to interact with short regions of dsRNA (13). Unexpectedly, we observed a substantial enrichment of an off-template G upstream of the first nucleotide of the 5′-UTR, which is compatible with the 7-methylguanosine cap structure previously observed with the antiviral cap-binding protein GEMIN5 (fig. S3) (38).

To further understand OAS1-mediated sensing, we assessed the transcriptome-wide binding of OAS1. Interestingly, the number of host transcripts associated with OAS1 was substantially higher in infected cells when compared to mock controls (Fig. 3J,K). This suggests that OAS1 RNA-binding activity may be enhanced by SARS-CoV-2 infection. OAS1 interacted primarily with cellular RNAs that are highly structured, including snoRNAs, lncRNAs and the intronic regions of mRNAs (Fig. 3J). These data are compatible with the notion that OAS1 senses short stretches of dsRNA that are found in stem loops. We also observed that OAS1 interacted with several mitochondrial RNAs (fig. S3), which is consistent with the presence of dsRNA in the mitochondria that can induce innate immune responses (39).

To identify potential drivers of the high specificity of OAS1, we analysed all the binding sites present in cellular RNAs using a variety of approaches. Both MEME (40) and HOMER (41) consistently identified a prominent motif (UCUACGG) and HOMER detected 2 additional nucleotide signatures before (CG) and after (C) this motif. While the first U and GG were relatively conserved, the middle of this motif was more variable (Fig. 3L). Strikingly, three versions of the motifs identified in the host transcriptome were apparent in the 5′-UTR of SARS-CoV-2 RNA, suggesting OAS1 binds SL1 and SL2 simultaneously or that multiple OAS1 molecules bind to multiple sites in this region, either on the same or distinct RNAs (Fig. 3L). Since OAS1 is known to interact with dsRNA (13), we also applied GLAM2 (40), which allows identification of binding sites with gaps. The motifs identified by GLAM2 were longer and more heterogeneous. However, it was possible to distinguish two additional features, a UU…UG and a GA…AT (fig. S3E). These gapped motifs are similar to those previously identified in RNA substrates (42, 43). Indeed, the WWN_9_WG motif (43) is also present in SL1 and SL2, with the G equivalent to position 9 in Fig. 3L. When combined, structural context and sequence specificity could potentially explain the high degree of specificity exhibited by OAS1, which is likely necessary in order to avoid the inappropriate activation of RNase L.

## The antiviral activity instigated by OAS1 is highly specific

To further understand the specificity of OAS1-mediated sensing, we considered the ability of OAS1 to initiate inhibition of a panel of viruses that replicate via dsRNA intermediates within different subcellular compartments. We first confirmed that OAS1 was active against the more transmissible B.1.1.7 variant of SARS-CoV-2, which remained highly sensitive to OAS1 restriction (Fig. 3M). Intriguingly, when we examined three negative sense ssRNA viruses whose genome replication occurs in the cytosol (Indiana vesiculovirus (VSV), human respirovirus 3 (PIV-3) and human respiratory syncytial virus (RSV)), all were unaffected by OAS1 (Fig 3C,M). Similarly, influenza A viruses (possessing a segmented negative sense ssRNA genome) whose replication occurs in the nucleus, were completely resistant to OAS1 (Fig. 3M). In contrast, when we examined Cardiovirus A (EMCV), a positive-sense ssRNA virus whose genome replication occurs within replicative organelles and double-membrane vesicles (DMVs) (44), OAS1 restricted this virus by >100-fold (Fig. 3C, M) (45). This notable antiviral specificity could be driven by virus-specific OAS evasion/antagonism strategies (46), such as the evasion role proposed for IAV NS1 (47). However, because CoVs replicate in similar ER-derived membranous structures to EMCV (48–50), we also considered whether OAS1 might be a dsRNA sensor specifically targeted to membranous replicative organelles in infected cells.

## C-terminal prenylation is necessary for OAS1 to initiate a block to SARS-CoV-2

**Figure 4:**
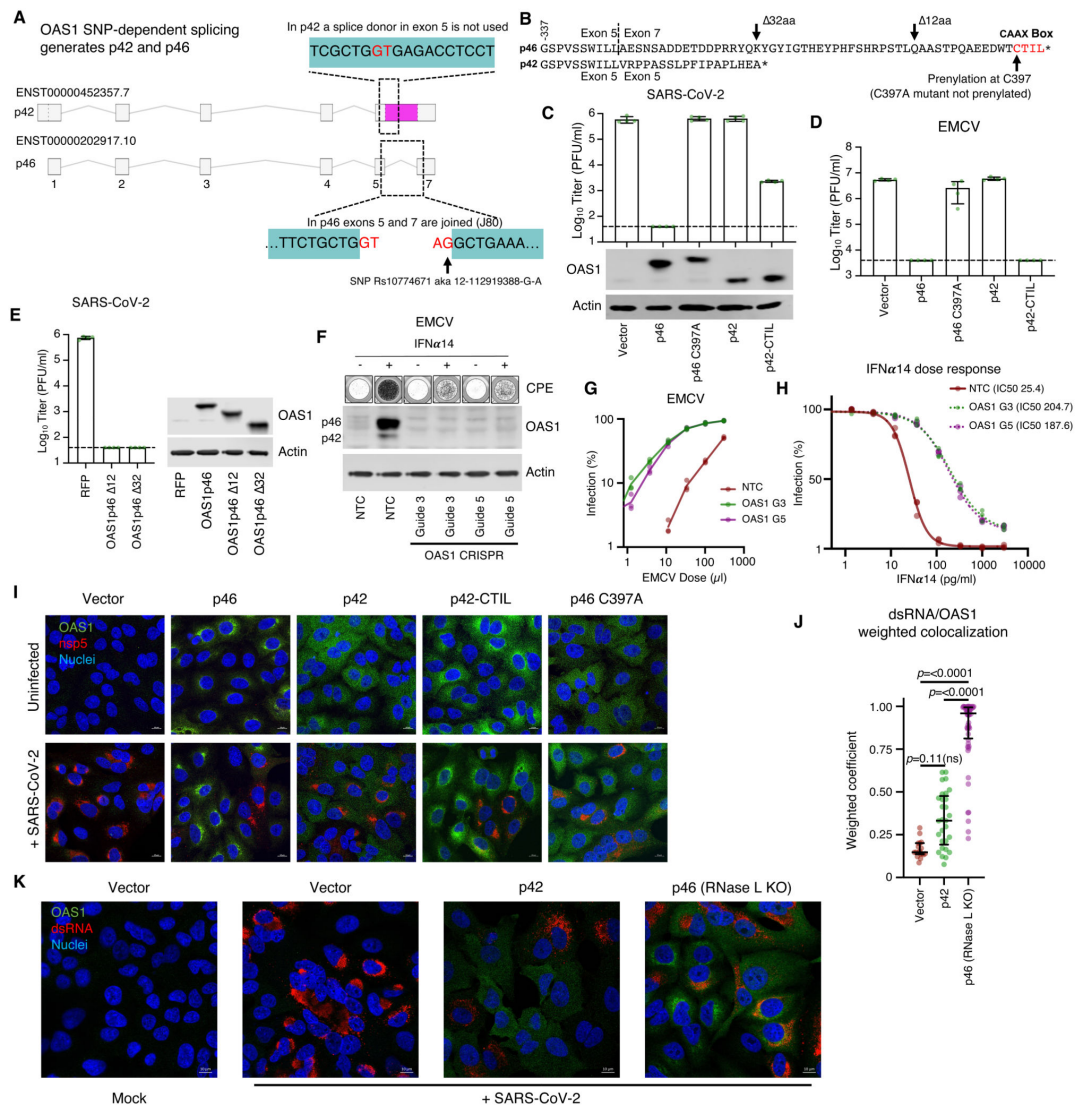
OAS1 isoforms have differential antiviral activity as determined by C-terminal prenylation. (A) Schematic representation of OAS1 splicing resulting in isoforms p42 and p46. The area shaded in pink is exonic in p42 and intronic in p46. (B) Protein sequence alignment of the p46 and p42 isoforms, indicating the CAAX box prenylation signal in p46 and locations of modifications made in this work. (C) SARS-CoV-2 infectious titer (PFU) on AAT cells expressing the OAS1 isoforms p46, not prenylated p46 (p46 C397A), p42 or prenylated p42 (p42CTIL) or a vector control. Protein expression analysis of the levels of isoforms and mutants is shown by western blot. (D) EMCV infectious titer on the cells from (B) as determined by plaque assay (PFU/ml). (E) SARS-CoV-2 infectious titer (PFU/ml) on AAT cells expressing OAS1 p46 or the p46 C-terminal truncations OAS1 p46 Δ12 and OAS1 p46 Δ32. The level of expression is shown by western blotting. (F) EMCV replication in HAT cells with reduced OAS1 expression using two different lentiviral vector-derived CRISPR guides and one non-targeting control (NTC) guide. Well clearance at 24 hpi was assessed in the presence or absence of pretreatment with 1000 pg/ml IFN***α***14 (typical wells shown in top panel), and level of OAS1 KO was assessed by western blotting. (G) EMCV infectious virus titration (based on % well clearance) in HAT cells whose OAS1 expression was reduced using two different OAS1 KO guides, compared to a NTC. (H) EMCV infection (% well clearance) after pretreatment of various doses of IFN***α***14 in same cells as Fig 4G. (I) Representative immunofluorescence on cells from (C) infected with SARS-CoV-2 isolate CVR-GLA-1 at MOI 0.5 for 24h followed by staining with anti-OAS1 (green) and anti-SARS-CoV-2-nsp5 (red) antibodies and nuclear Hoechst stain (blue). Contrast was reduced in the p46 sample to prevent oversaturation in the green channel due to particularly strong perinuclear concentration. Representative cells from one out of three independently performed experiments are depicted. (J) Quantification of colocalization of dsRNA with OAS1 (weighted colocalization coefficient) in infected cells represented in Fig 4K. Each data point represents a distinct region of interest encompassing an individual cell from one representative experiment. (K) Representative immunofluorescence on AAT cells modified with a vector control, OAS1 p42 or OAS1 p46 in the presence of RNase L KO, infected or mock treated with SARS-CoV-2 isolate GLA-1 at MOI 0.5 for 24h, followed by staining with anti-OAS1 (green) and anti-dsRNA (red) antibodies and nuclear Hoechst stain (blue). Representative cells from one out of two independently performed experiments are depicted.

5 In humans, OAS1 protein is expressed as two major forms designated p46 and p42. The longer p46 isoform (present in the screening library and used in Figures 1-3) is generated by alternative splicing to an exon downstream of the terminal exon utilized by the p42 isoform (Fig. 4A,B). Although all human genotypes possess the exon that completes the transcript encoding p46, an intronic SNP (Rs10774671 aka 12-112919388-G-A) determines OAS1 exon usage. Alleles with a G at this SNP (G alleles) specify expression of the p46 isoform and some p42, whereas alleles with A at this position predominantly encode the p42 isoform and cannot express the p46 isoform (51–53). Individuals with G alleles are more resistant to West Nile virus infection (54) and respond better to IFN therapy following hepatitis C infection (55). Importantly, G alleles are also associated with protection against severe COVID-19 disease (1, 22, 24, 25). We thus determined whether p42 possessed the same anti-SARS-CoV-2 activity as p46. Remarkably, the p42 isoform, which is the most common isoform in humans (~61% of alleles) possessed no detectable anti-SARS-CoV-2 activity (Fig. 4C). Although differential basal enzymatic activity was initially proposed to underlie the divergent antiviral potential of p46 and p42 OAS1 (51), this effect is likely due to expression level, as p42 and p46 possess similar catalytic activities (56). Examination of the C-terminus of p46 reveals it encodes a canonical CAAX-box prenylation signal (CTIL) that is absent from the p42 variant (Fig. 4B), and is predicted to be geranylgeranylated (57). Indeed, prenylation of OAS1 was proposed to alter the subcellular localization of OAS1, perhaps influencing mitochondrial respiration (58). We therefore hypothesized that prenylated OAS1 is targeted to membranous viral replicative organelles and facilitates the sensing of CoV dsRNA (and perhaps many divergent picornaviruses, arteriviruses, caliciviruses and flaviviruses that also utilize replicative organelles). To test this, we introduced a point mutation into the p46 isoform which prevented its prenylation (C397A), and this completely ablated the antiviral activity of p46 (Fig. 4C). Similarly, appending a four amino acid CAAX-box (CTIL) to the C-terminus of the p42 isoform conferred substantial antiviral activity to the inactive p42 form, reducing the ability of SARS-CoV-2 to form plaques by more than 100-fold (Fig. 4C). Thus, prenylation of OAS1 appears necessary for dsRNA sensing of SARS-CoV-2. A nearly identical picture emerged using EMCV (Fig. 4D). While prenylated p46 and p42-CTIL reduced EMCV plaque formation by >100-fold, nonprenylated p42 or p46 C397A possessed no anti-EMCV activity. Importantly, this antiviral activity of p46 and p42-CTIL was highly specific and did not inhibit the ability of VSV to form plaques in parallel experiments (fig. S4A). Whilst potent, prenylated p42-CTIL instigated a weaker block to SARS-CoV-2 than p46 (Fig. 4C), we therefore considered whether additional determinants resided in the C-terminal region. A relatively short 18 aa fragment of the p46 C-terminal (the same length as p42-CTIL) was indistinguishable from p46 in its ability to initiate a block to SARS-CoV-2 (Fig. 4E), indicating that most of the 54 aa C-terminus of p46 is dispensable for efficient inhibition of SARS-CoV-2.

## Endogenous OAS1 makes a substantial contribution to the antiviral state

To evaluate the contribution that OAS1 makes to the antiviral state, we ablated OAS1 expression in HT1080-ACE2-TMPRSS2 cells, which are heterozygous at Rs10774671 and predominantly express p46 following IFN stimulation (Fig. 4F) (29). We then ablated OAS1 expression using CRISPR-Cas9 and examined the ability of type I IFN to inhibit EMCV replication (Fig. 4F). Notably, IFN pretreatment inhibited approximately 20 times more virus replication in the presence of OAS1 (Fig. 4G). In the absence of OAS1 expression, approximately 7 times as much IFN was required to inhibit viral replication than when OAS1 was present (IC_50_ of 25 pg/ml in the control versus an IC_50_ of 188 pg/ml in the OAS1 KO) (Fig. 4H). Thus, the antiviral inhibition initiated by endogenous OAS1 can play a major role in generating an antiviral state. Similar unpublished experiments with SARS-CoV-2 were attempted but were less conclusive, likely because SARS-CoV-2 was so profoundly inhibited by type I IFNs. Indeed, IFN treatment reduced viral replication by several orders of magnitude in A549 cells that cannot express prenylated OAS1 (29) (fig. S1). This indicates that in the interferon-stimulated cell, prenylated OAS1 is likely only one of multiple ISGs that mediate the potent inhibition of SARS-CoV-2.

## Prenylated OAS1 colocalizes with viral dsRNA

Because SARS-CoV-2 replication, which uses dsRNA intermediates, occurs within membranous replicative organelles, we next considered whether prenylation localizes OAS1 to these replicative organelles. Prenylated p46 and p42-CTIL localized to membranous perinuclear structures reminiscent of the ER (Fig. 4I), whereas nonprenylated p42 and p46 C397A were diffusely distributed (Fig. 4I). To determine whether prenylated OAS1 localized to SARS-CoV-2 replicative organelles, we co-stained infected cells with the viral nsp5 (8). However, the p46 block was sufficiently strong to prevent formation of nsp5+ replicative structures, which were only visible in clusters of cells expressing low levels of p46 (Fig. 4I). To overcome this, we imaged infected OAS1 expressing cells, whose RNase L expression was disrupted using CRISPR-Cas9. Relieving the block to SARS-CoV-2 replication imposed by OAS1 facilitated the visualization of SARS-CoV-2 replicative structures (Fig. 4I and fig. S4B). While OAS1 expression was enriched in close proximity to nsp5, these proteins did not appear to colocalize. We thus examined the colocalization of OAS1 and the corresponding dsRNA PAMP (pathogen associated molecular pattern). In the absence of RNase L, SARS-CoV-2 dsRNA colocalized with prenylated OAS1 (Fig. 4J,K). In contrast, dsRNA detection overlapped poorly with nonprenylated p42 (Fig. 4J,K, fig S4C). Considered alongside the iCLIP experiments (Fig. 3I-K, fig. S3), these data indicate that prenylation targets OAS1 to sites rich in viral dsRNA, which are probably the SARS-CoV-2 replicative organelles. Once in the right place, OAS1 binds to dsRNA structures in the SARS-CoV-2 5′-UTR and initiates a potent block to SARS-CoV-2 replication.

## Prenylated OAS1 is associated with less severe COVID-19

**Figure 5:**
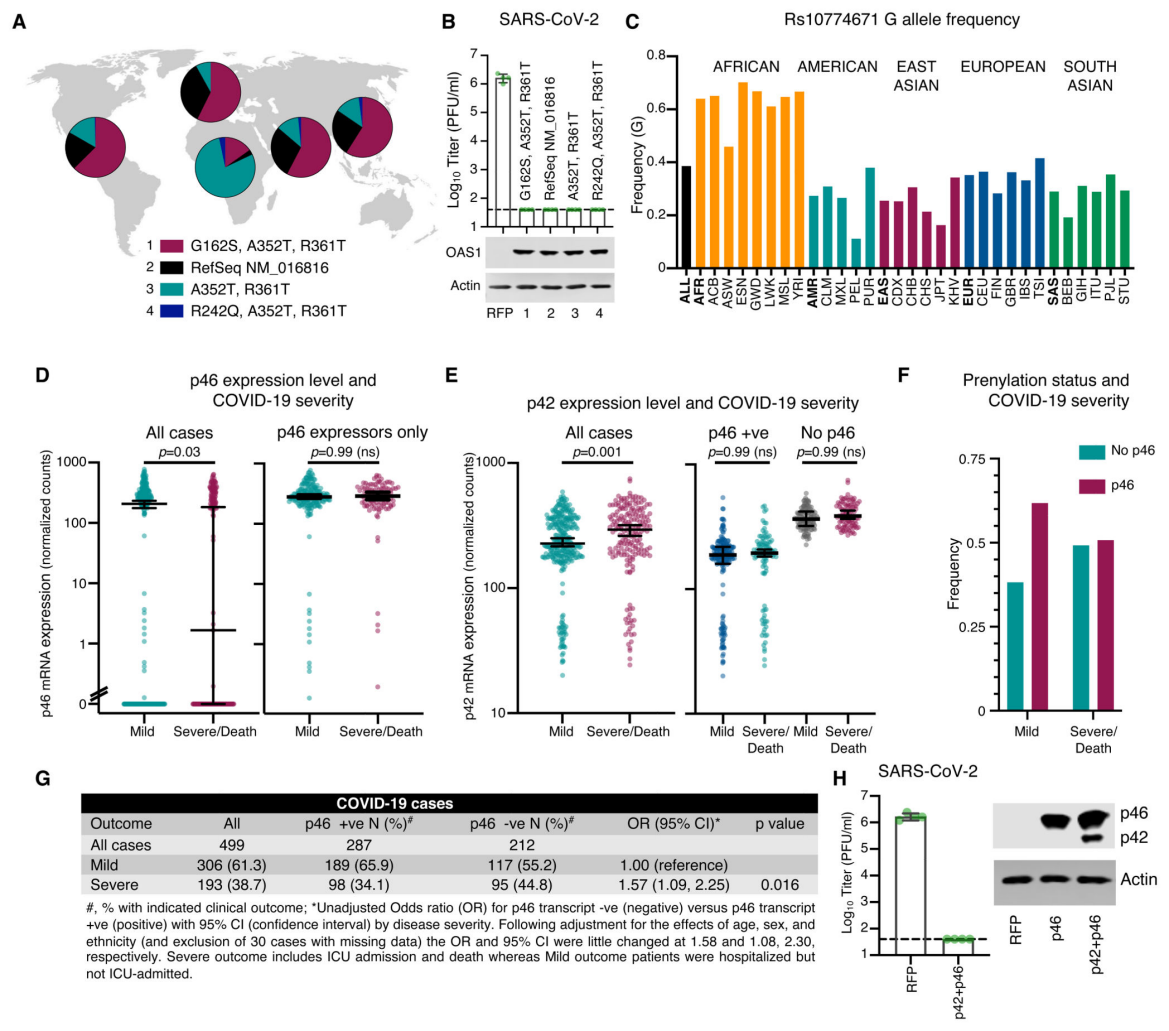
Prenylated OAS1 protects against severe COVID-19. (A) Allelic frequencies of the most common circulating p46 variants of OAS1 displayed by region. (B) Infectious titers of SARS-CoV-2 CVR-GLA-1 (PFU/ml) were determined on AAT cells modified to express each human p46 OAS1 variant. OAS1 expression was monitored using western blotting (lower panels). (C) Frequency of alleles with G at Rs10774671 in different human populations (1000 genomes project). The population names are expanded in the materials and methods. (D) Transcript abundance of the p46 isoform (encoding prenylated OAS1), determined using JunctionSeq analysis (J080) of RNA-seq data from whole blood from infected patients with mild (hospitalized but not ICU-admitted) or severe/lethal (ICU-admitted and/or death) COVID-19. (E) Transcript abundance of the p42 isoform (E037) determined as in E. For D and E, significance was determined using a Mann-Whitney U test except where multiple comparisons were made, (righthand panel of E) and then a Kruskal-Wallis rank sum test was used. All four comparisons not highlighted were significant (p <0.0001). (F) Prenylation status (p46 −ve or +ve), determined by the presence or absence of p46 transcript (from D) in mild and severe COVID-19. (G) Tabulated odds ratios and 95% confidence intervals of the data presented in D and F. (H) SARS-CoV-2 infectious titer on AAT cells expressing the OAS1 isoforms p46 or p46 and p42. Isoform expression level (western blot) is also shown.

The realization that prenylation is essential for OAS1-mediated sensing of SARS-CoV-2 allowed us to examine the transcriptome of infected patients and investigate whether there is a link between the expression of prenylated OAS1 and SARS-CoV-2 disease progression. Importantly, the 4 most common p46 variants (Fig. 5A), all conferred protection against SARS-CoV-2 infection when exogenously expressed (Fig. 5B). Because each p46 variant possessed antiviral activity (i.e. was not confined to a single haplotype), we examined the p46 splice junction directly (Fig. 4A) to assess whether expression of p46-encoding mRNA, as opposed to the presence or absence of SNPs (1), significantly influenced the severity of COVID-19. Importantly, the frequency of the Rs10774671 G SNP that governs the expression of p46 varies between ~11% (population: Peruvian, in Lima) and ~70% (population: Esan, in Nigeria) (Fig. 5C) and could have a major influence on the susceptibility of different populations to severe COVID-19.

Although the p46 transcript encodes 54 C-terminal amino acids (aa) that are not part of the p42 protein (Fig. 4B), individuals homozygous for A at Rs10774671 (AA) can form splice junctions 1 nucleotide downstream of the p46 splice junction. We therefore confirmed that we could reliably detect the absence of p46 in RNA-seq data derived from cells with an AA genotype. We used JunctionSeq to examine all OAS1 transcript junctions (annotated on Ensembl) in these AA cells in the presence or absence of IFN treatment (fig. S5A-C). Accordingly, we were unable to detect the specific junction (J080) between exons 5 and 7 that specifies the expression of prenylated OAS1 in these cells (fig. S5A-C).

We therefore applied this method to RNA-seq data from 499 hospitalized UK COVID-19 patients with known disease outcomes (ISARIC4C) (fig. S6A-C). We defined severe outcomes as intensive care unit (ICU) admission and/or death, whereas mild outcome patients were hospitalized but not ICU-admitted. All patients expressed detectable OAS1 but 42.5% of individuals (212/499) did not express p46. Remarkably, the absence of prenylated OAS1 was associated with more severe disease (Fig. 5D-G). Specifically, the median transcript abundance of p46 was over 100-fold lower in the severe COVID-19 group (Fig. 5D). This difference was entirely driven by the over-representation of patients in the severe COVID-19 group who did not express any prenylated OAS1. p46 mRNA levels were almost identical in individuals that expressed prenylated OAS1, regardless of whether they experienced mild or severe COVID-19 (Fig. 5D). Similarly, increased p42 expression was also associated with more severe COVID-19 (Fig. 5E). However, this association appeared to be a surrogate measurement of p46 expression, as patients who did not express any prenylated OAS1 expressed substantially higher levels of p42 (Fig. 5E). Crucially, no difference in p42 expression was apparent once the ability to express p46 was considered (Fig. 5E). Importantly, patients lacking the p46 transcript were more frequently observed in the severe disease group (Fig. 5F) and were significantly more likely to experience severe disease (95/212, 44.8%) compared to those expressing p46 (98/287, 34.1%) (unadjusted Odds Ratio (OR)=1.57, 95% CI 1.09, 2.25; following adjustment for age, sex, and ethnicity and exclusion of 30 cases with missing data OR=1.58, 95% CI 1.08, 2.30) (Fig. 5G). Death was also more frequent in these patients (34/212, 16% versus 34/287, 11.8%) with the effect size similar to that for disease severity, but these differences were not statistically significant (unadjusted OR 1.42, 95% CI 0.85, 2.37). Because higher p42 expression was associated with increased disease severity (Fig. 5E), we examined whether p42 influenced the ability of p46 to inhibit SARS-CoV-2. In accordance with the lack of inhibition observed in Fig. 4C, p42 did not substantially blunt the ability of p46 to initiate a block to SARS-CoV-2 (Fig. 5H). Again, this is consistent with the association of high p42 expression with severe COVID-19 being mechanistically underpinned by an absence of p46.

Previous studies have identified an OAS1 haplotype that was inherited from Neanderthals and that was associated with reduced susceptibility to SARS-CoV-2 infection and protection from severe COVID-19 (22, 24). Our evaluation of OAS1 antiviral activity in vitro, combined with our analysis of OAS1 transcripts in patient cohorts, indicates that the protective Neanderthal OAS1 haplotype (22, 24) likely prevents severe disease through specifying the expression of prenylated OAS1, which directs dsRNA sensing to the sites of SARS-CoV-2 replication. Indeed, through combining multiple studies, Huffman *et al.*, also concluded that the Rs10774671 SNP is responsible for the protection conferred by the Neanderthal haplotype (59).

## An ancient retrotransposition event ablated OAS1 prenylation in horseshoe bats

**Figure 6:**
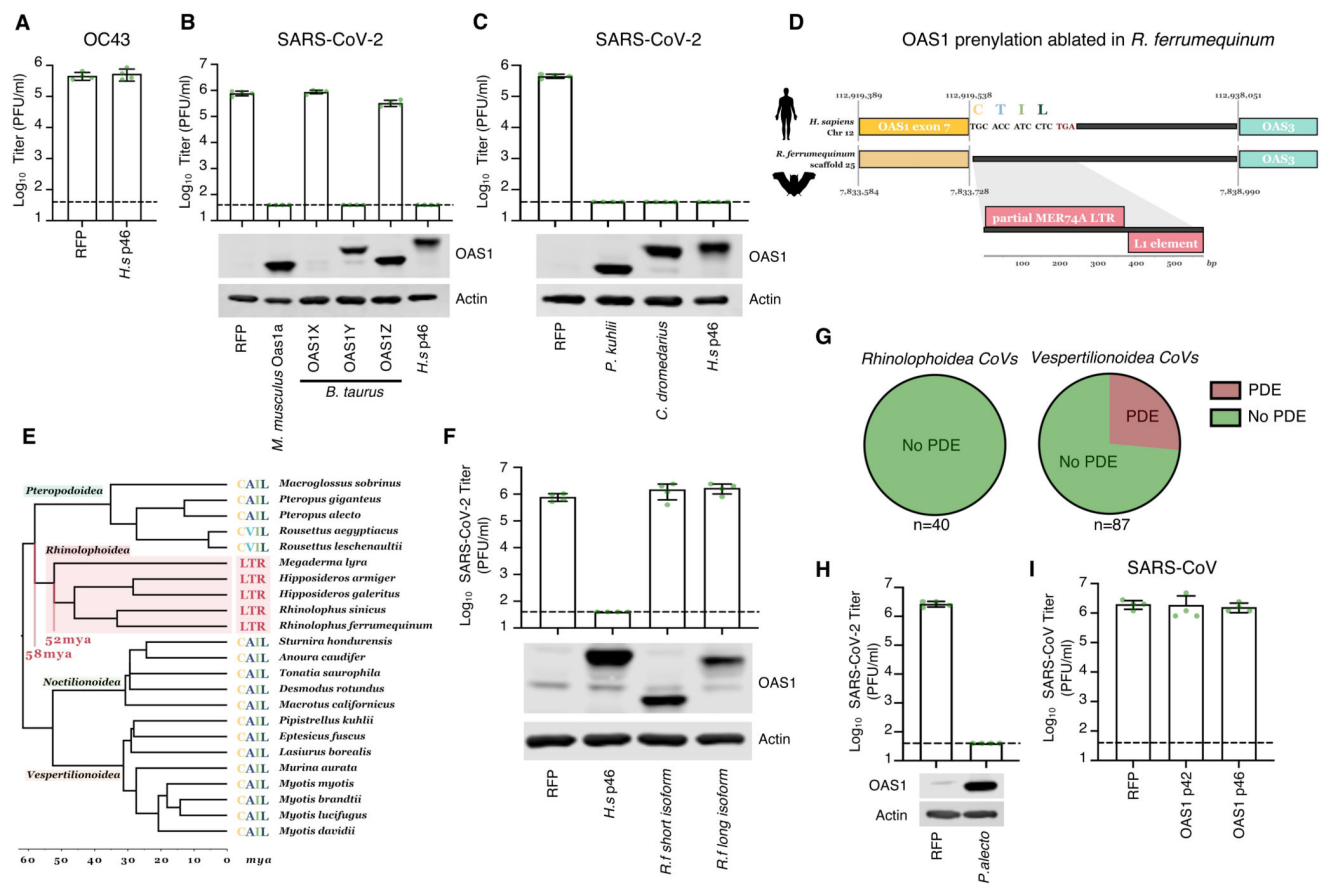
Retrotransposition at the OAS1 locus has ablated the CAAX-box prenylation signal in *Rhinolophoidea*. (A) Infectious titers of OC43 (PFU/ml) were determined on AAT cells modified to express OAS1 from humans (*H.s p46*). (B) Infectious titers of SARS-CoV-2 CVR-GLA-1 (8) (PFU/ml) were determined on AAT cells modified to express Oas1a from mouse (*M. musculus*), OAS proteins from cows (*B. taurus) and* human p46 (*H.s* p46). OAS1 expression was monitored by western blotting (lower panels). (C) Infectious titers of SARS-CoV-2 (PFU/ml) were determined on AAT cells modified to express OAS1 from pipistrellus bats (*P. khulii*), dromedary camels (*C. dromedarius*) and human p46 (*H.s* p46). OAS1 expression was monitored by western blotting. (D) Schematic of genome synteny between the human OAS1 exon 7 locus (yellow) and the *R. ferrumequinum* genome. The exact syntenic sequence coordinates are annotated for the start of OAS1 exon 7, the start of the CAAX box encoding sequence and the start of the upstream gene locus, OAS3 (blue). Transposable element hits on the 580bp non-syntenic region in the *R. ferrumequinum* genome are shown in the zoomed in inset. Non-coding regions are shown in black. Note that the schematic is not to scale. (E) Dated phylogeny (retrieved from timetree; www.timetree.org (101) of bat species with a confirmed LTR insertion in the OAS1 locus or a CAAX box encoding sequence present in the same scaffold as their OAS1 locus. Clades are labelled by superfamily, species names and CAAX sequence (or LTR) are annotated next to the tree tips. The approximate time period during which the LTR insertion took place is annotated in red. (F) Infectious titers of SARS-CoV-2 CVR-GLA-1 (PFU/ml) were determined on AAT cells modified to express OAS1 from humans (*H.s* p46) and horseshoe bats (*R.f*) using both NCBI and Ensembl database entries. OAS1 expression was monitored by western blotting. (G) Pie charts of CoVs from *Rhinolophoidea* and *Vespertilionoidea* binned according to whether they are known or predicted to encode a phosphodiesterase (PDE) OAS antagonist. (H) Infectious titers of SARS-CoV-2 (PFU/ml) were determined on AAT cells modified to express OAS1 from the black fruit bat (*P. alecto*). OAS1 expression was monitored by western blotting. (I) Infectious titers of SARS-CoV (PFU/ml) were determined on AAT cells modified to express human OAS1 p42 or p46.

Every species has a unique repertoire of genome-encoded antiviral defenses (26). As the differential splicing of p46 and p42 isoforms is poorly characterized beyond primates, it was previously difficult to investigate the protection conferred by p46 in non-human species. The realization that prenylation can be essential for antiviral activity allowed us to investigate this aspect of OAS1 biology beyond humans. For example, many coronaviruses encode phosphodiesterases (PDEs) that degrade 2-5A and antagonize the OAS system (60). The human betacoronavirus OC43 encodes such a phosphodiesterase (NS2) (61) and accordingly, we found that prenylated OAS1 did not inhibit OC43 (Fig. 6A). Similarly, the Middle East respiratory syndrome-related CoV (MERS-CoV) also encodes a PDE (NS4b) capable of antagonizing the OAS system (62). We hypothesized that the reservoir species of OC43 and MERS-CoV encode OAS proteins that can initiate a block to CoV replication. OC43 likely originated in a murine host (63) and entered human populations via a cross-species transmission from cows (64). Examination of mouse and cow OAS1 sequences identified 8 murine paralogs, three of which have CAAX boxes (Oas1a, Oas1f and Oas1g), and 3 bovine paralogues, one of which has a CAAX box (OAS1Y). In accordance with Fig. 4, prenylated murine OAS1a and prenylated bovine OAS1Y both conferred potent anti-SARS-CoV-2 activity, whereas nonprenylated bovine OAS1Z did not (Fig. 6B, note that we were unable to confirm efficient expression of bovine OAS1X using polyclonal antibody raised to human OAS1).

Close relatives of MERS-CoV have been identified in bats (such as *Pipistrellus kuhlii*) (65) and MERS-CoV entered human populations following transmission from dromedary camels (*Camelus dromedarius*) (66, 67). Accordingly, OAS1 from *P. kuhlii* and *C. dromedarius* have CAAX boxes and both instigated potent blocks to SARS-CoV-2 (Fig. 6C). Notably, the *P. kuhlii* C-terminus is shorter than human p42, reinforcing the notion that most of the extended C-terminus of p46 is not necessary for antiviral activity (fig. S7A). Crucially, this means that all the species believed to harbor either OC43 or MERS-CoV en route to emergence in humans express prenylated OAS1 proteins that could credibly have selected for the maintenance of PDE expression in these viruses.

The extreme sensitivity of SARS-CoV-2 to prenylated OAS1 also led us to determine whether horseshoe bats, the likely source of SARS-CoV-2, possess a prenylated OAS1 defense. There is a paucity of mRNA sequence data available for horseshoe bats and we were unable to find OAS1 database entries from the likely bat hosts of the precursors of SARS-CoV-2 (*Rhinolophus affinis* or *pusillus* (68)) and SARS-CoV (*Rhinolophus sinicus*). Thus, we analysed OAS1 transcripts from the greater horseshoe bat (*Rhinolophus ferrumequinum*). Interestingly, we were unable to find a transcript or exon encoding a prenylated OAS1 in *R. ferrumequinum*. Indeed, all Ensembl and NCBI database entries specified nonprenylated proteins. When we examined the genomic region where the prenylation signal should reside (based on synteny and homologous flanking sequences), retrotransposition of an LTR sequence was evident, and this ablated the CAAX-box motif, preventing the expression of prenylated anti-CoV OAS1 in these bats (Fig. 6D). We searched for this insertion in 44 available bat genome sequences and identified the same insertion only in members of the Rhinolophoidea superfamily (including Rhinolophus, Hipposideros and Megaderma species), indicating that this ancient retrotransposition insertion occurred ~58-52 million years ago (MYA), within this bat superfamily. In contrast, we could detect CAAX-box encoding syntenic sequences in members of all other bat taxa (Fig. 6E).

Based on the absence of prenylated OAS1 in Rhinolophoidea, we predicted that OAS1 from *Rhinolophus ferrumequinum* would be inactive against SARS-CoV-2. Accordingly, the best supported OAS1 isoforms from the greater horseshoe bat (fig. S7B) did not inhibit SARS-CoV-2 (Fig. 6F). Importantly, this means that membrane-associated, prenylated, p46-like, OAS1 dsRNA sensing has been ablated in the presumed bat reservoirs of SARS-CoV and SARS-CoV-2, and this may contribute to horseshoe bats being such prolific reservoir hosts of Sarbecoviruses. The absence of pressure to evade prenylated OAS1 (in horseshoe bats) may have left SARS-CoV-2 particularly sensitive to this defense (when subsequently encountered in human populations).

Considering the lack of prenylated OAS1 in Rhinolophoidea and the ability of coronavirus PDEs to antagonize this pattern recognition pathway, we sought to examine whether PDE-encoding coronaviruses infect horseshoe bats. Given the variability in coronavirus encoded PDEs (NS4b in Merbecoviruses and NS2 in Embecoviruses (61, 62)), we developed a custom HMM protein profile using NS4b, NS2, the mammalian PDE AKAP7 (69) and rotavirus A VP3 (70). We screened for PDEs through all putative open reading frames (ORFs) of all published Coronaviridae genomes. This method should capture previously unannotated or undescribed PDEs. Although the available sequence data set is likely biased by sampling, we could not identify PDEs in any known coronaviruses from Rhinolophoidea. In fact, all the bat coronaviruses identified as encoding PDEs were from bats in the Vespertilionoidea superfamily (whose prenylated OAS1 is intact) (Fig. 6G). Whilst there is a striking absence of PDEs in the CoVs that circulate in horseshoe bats, an absence of PDEs does not necessarily imply an absence of anti-CoV OAS proteins in the relevant host. Many potential strategies exist to evade or antagonize the OAS system (46) and we note that we also did not identify PDEs in CoVs sampled from Pteropodoidea. To confirm that prenylated OAS1 from megabats have anti-CoV potential, we observed that OAS1 from *P. alecto* could instigate a potent block to SARS-CoV-2 (Fig 6H).

Prior to the COVID-19 pandemic, SARS-CoV emerged in humans in 2003. Close relatives of SARS-CoV circulate in *R. sinicus* (71), and SARS-CoV was transmitted to humans via an intermediate species, widely believed to be civets (72). Surprisingly, when we considered the ability of human OAS1 to inhibit an isolate of SARS-CoV, this virus was completely resistant to human p46 and p42 (Fig. 6I). Because the retrotransposition event that ablates the prenylation sequence was confirmed in *R. sinicus* (Fig. 6E), we speculate that SARS-CoV may have acquired the ability to evade or antagonize OAS1 during circulation in an intermediate species, or in human populations.

## Discussion

Viruses tend to rapidly adapt to new host species and even SARS-CoV-2, a ‘generalist’ virus (73), has likely adapted to replicate in the animal reservoir(s) in which it circulated prior to emergence in humans. Cross-species transmission exposed SARS-CoV-2 to a new repertoire of human antiviral defenses, some of which SARS-CoV-2 may not have encountered before. Prenylated OAS1 may be an example of such a defense, with prenylated OAS1 being targeted to endomembranous structures where it initiates potent anti-SARS-CoV-2 activity in vitro.

Importantly, hospitalized COVID-19 patients lacking the p46 transcript had worse clinical outcomes than those who expressed prenylated OAS1. Severe disease was significantly more frequent with ICU admission or death being approximately 1.6 times more likely in these patients. The increased odds of death were similarly raised among patients lacking p46. However, the number of deaths in this cohort was relatively small and a larger study would be needed to provide enough power to determine whether a lack of p46 transcript is also associated with increased mortality. In addition, we could not detect an association between disease severity and p46 transcript abundance in individuals who express p46. This could reflect the fact that expression level is less important for an enzyme (because catalysis greatly amplifies pattern recognition). It should also be considered that p46 expression in whole blood may not recapitulate expression differences at the sites of viral replication, or important differences present early in infection (before hospitalization). Along these lines, OAS1 levels have been linked to the severity of COVID-19 in other work (22). Although prenylated OAS1 tips the balance in favor of the host in a significant number of people, OAS1 is just one component of a ‘successful’ immune response and it is likely that multiple ISGs (alongside many other factors) influence the outcome of SARS-CoV-2 infection.

Considering the apparent lack of antiviral activity of OAS1 p42, it is notable that the p42-encoding alleles predominate and are more common in all human populations (apart from people of African descent). The geographical variation in the frequency of alleles encoding prenylated OAS1, such as the high frequency observed in some African populations, could potentially influence the spread and severity of COVID-19. It is currently difficult to reconcile a potential role for p46 in reducing the impact of COVID-19 in some African countries (59, 74) with the increased disease severity observed in people of African descent (75). We note that none of our observations refute the possibility that p42 enhances the anti-SARS-CoV-2 activity of p46. It is possible that p42 may be more beneficial than p46 in some contexts, potentially targeting viruses that don’t utilize replicative organelles. Alternatively, the p46 variant may have been selected against, possibly because it is deleterious in the absence of specific viral infections (56, 76). In support of this notion, the catalytic activity of OAS1 has been lost entirely in some species (56). Because p46 may reduce the susceptibility to SARS-CoV-2 infection (22), the billions of individuals that are unable to express prenylated OAS1 may make humans relatively vulnerable to the direct cross-species transmission of Sarbecoviruses from horseshoe bats.

There is currently great interest in identifying the biological characteristics of bats that might predispose them to be reservoirs of circulating viruses (77) and much work has focused on innate immunity (78). It is important to be cautious when generalizing about bats as each species possesses unique innate immune features (26). Bats are an extraordinarily diverse order (>1400 species) (79) and individual bat species may not be any more likely to act as viral reservoirs than other species (80). Nevertheless, it is striking that horseshoe bats not only lack prenylated OAS1-mediated dsRNA sensing but have a reduced ability to sense cytosolic DNA through STING (81). It is tempting to speculate that multiple defects in pathogen recognition may make horseshoe bats particularly good virus reservoirs. However, this requires resolution regarding why innate immune defects might promote tolerance in bats while promoting pathogenesis in humans.

The endomembrane targeting of prenylated OAS1 enables the potential sensing of a diverse spectrum of viruses. For example, multiple viruses that utilize replicative organelles, including Hepatitis C Virus (82), Alphaarterivirus equid (EAV) (83) and Betaarterivirus suid 1 (PRRSV) (84), are inhibited by OAS1. However, identification of the sites in viral and host RNAs that were bound by OAS1 underscored how selective a sensor OAS1 is. Few host RNAs were bound by OAS1 and despite replicating via a dsRNA intermediate, most sites in the SARS-CoV-2 genome escaped detection by OAS1. It appears that multiple layers of antiviral specificity exist for prenylated OAS1, and OAS1 likely only recognizes a specific subset of dsRNA sequences that occur close to endomembranes. The remarkable target specificity of OAS1 is likely necessary because OAS1 senses relatively short stretches (~18 bp) of dsRNA (13) and a less discerning sensor would be inappropriately activated by cellular RNAs.

The ability of SARS-CoV to escape inhibition by human p46 highlights that while many viruses might conceivably be targeted, evasion and antagonism strategies (46) mean that sensitivity for every virus must be considered on a case-by-case basis. In accordance with this, we note that although SARS-CoV diverges from SARS-CoV-2 at several positions within the 5′-UTR, it is currently unclear whether this is the strategy that enables SARS-CoV to escape from OAS1-mediated sensing.

Prenylated OAS1 has contributed to the prevention of severe COVID-19 in a substantial fraction of infected individuals (likely measured in ‘hundreds of thousands’ in the UK alone). With the continued emergence of SARS-CoV-2 variants of concern, it will be important to remain vigilant. The chance that recombinant acquisition of a PDE gene (from a coinfecting virus or host gene (69, 85)) or repeated selection against SL1 and SL2 in the 5′-UTR could enable escape from this OAS1 defense, and increase the pathogenicity of SARS-CoV-2. This reinforces the need to pay close attention to the phenotypic properties of emerging SARS-CoV-2 variants.

## Materials and Methods

### Cell lines, plasmids, and viruses

All cells were maintained in Dulbecco’s modified Eagle’s medium (DMEM) supplemented with 9% FCS and 10 μg/ml gentamicin unless otherwise stated. A549-ACE2-TMPRSS2 (‘AAT’) and VeroE6-ACE2-TMPRSS2 (‘VAT’) cells have been described previously (8). HEK-293T cells were propagated from lab stocks, Vero E6 cells were a generous gift of Prof. Michele Bouloy, and A549-Npro cells were a kind gift of Prof. Richard E. Randall. HT1080 cells were a kind gift of Prof. Stuart Neil and were modified to overexpress human ACE2 and TMPRSS2 (referred to as ‘HAT’ cells) and were transduced as described previously (8). Calu-3 cells were a generous gift from Prof. Paul J Lehner and were maintained in MEM supplemented with 10% FCS, 2 mM glutamine, 2 mM sodium pyruvate and 100 μM non-essential amino acids.

The SARS-CoV-2 viruses CVR-GLA-1, England/02/2020 and SARS-CoV-2-ZsGreen have been described previously (8). SynSARS-CoV-2-eGFP was a kind gift from Prof. Volker Thiel (11). SARS-CoV-2 lineage B.1.1.7 isolate ‘212’ was isolated from a clinical sample (kind gift of Prof. Wendy Barclay). Indiana vesiculovirus (VSV) was a kind gift of Dr. Megan Stanifer (86). Influenza A viruses A/Puerto Rico/8/1934 (H1N1) and A/mallard/Netherlands/10-Cam/1999(H1N1) were rescued from reverse genetics systems (a kind gift from Prof. Ron Fouchier, and Prof. Laurence Tiley, respectively) as described previously (87, 88). Human respirovirus 3 with GFP (PIV3-GFP) was purchased from ViraTree. Human respiratory syncytial virus expressing GFP (RSV-GFP) was a kind gift from Prof. Peter Collins (89). Cardiovirus A (EMCV) was a kind gift from Dr. Connor Bamford. Betacoronavirus OC43 (ATCC VR-1558) was purchased from ATCC and propagated on VAT cells (8). SARS-CoV virus isolate (HKU39849, GenBank: AY278491.2) was a kind gift of Prof. Malik Peiris and supplied by Prof. Bart Haagmans.

### Retroviral vectors and cell modification

The lentiviral vector pSCRPSY (KT368137.1) has been previously described (5). pLV-EF1a-IRES-Puro (Addgene plasmid # 85132) or pLV-EF1a-IRES-Blast (Addgene plasmid #85133) were modified by PCR amplifying the TagRFP ORF (using pSCRPSY as template) flanked by directional *SfiI* sites, which were further flanked by *BamHI* and EcoRI restriction sites (forward oligo: 5′-CTC TCG GAT CCG GCC GAG AGG GCC ATG AGC GAG CTG ATT AAG-3′ and reverse oligo: 5′-CTC TCG AAT TCG GCC AGA GAG GCC TCA CTT GTG CCC CAG-3′) and the BamHI-EcoRI fragment was subcloned into the vectors to create the modified pLV-EF1a-IRES-Puro-S*fi*I-TagRFP or pLV-EF1a-IRES-Blast-S*fi*I-TagRFP constructs. The cDNA corresponding to the ORFs of the following OAS genes (GenBank accession number): OAS1p46 (NM_016816), human OAS3 (NM_006187), mouse Oas1A (NM_145211), bovine OAS1X (NM_178108), bovine OAS1Y (NM_001040606), bovine OAS1Z (AY650038), *Pipistrellus kuhlii* (XM_036409709.1), *Camelus dromedarius* (XM_031443284), *Rhinolophus* *ferrumequinum* OAS1 (short isoform: XM_033097132 / long isoform: ENSRFET00010016745), *Pteropus alecto* (NM_001290162), were synthesized as gene-blocks with flanking S*fi*I sites (IDT DNA), and the S*fi*I fragment was subcloned into the modified pLV-EF1a-IRES-Puro-S*fi*I plasmid. To generate the human OAS1p42 sequence (in accordance with GenBank accession NM_002534), OAS1p46-C397A, and OAS1p42-CTIL sequences, the pLV-S*fi*I-OAS1p46 lentiviral vector plasmid was modified by overlap extension PCR (using primer pair 5′- CTC TCT GGC CGA GAG GGC CAT GAT GGA TCT CAG AAA TAC CCC AG-3′ and 5′- TCT CTC GGC CAG AGA GGC CTC AAG CTT CAT GGA GAG GGG CAG GGA TGA ATG GCA GGG AGG AAG CAG GAG GTC TCA CCA GCA GAA TCC AGG AGC TCA CTG GG-3′ for OAS1p42, primer pair 5′- CTC TCT GGC CGA GAG GGC CAT GAT GGA TCT CAG AAA TAC CCC AG -3′ and 5′- TCT CTC GGC CAG AGA GGC CTC AGA GGA TGG TGG CGG TCC AGT CCT CTT CTG CCT GTG GG -3′ for OAS1p46-C397A, and primer pair 5′- CTC TCT GGC CGA GAG GGC CAT GAT GGA TCT CAG AAA TAC CCC AG -3′ and 5′- TCT CTC GGC CAG AGA GGC CTC AGA GGA TGG TGC AAG CTT CAT GGA GAG GGG CAG GGA TGA ATG GCA GGG AGG AAG CAG GAG GTC TCA CCA GCA GAA TCC AGG AGC TC ACT GGG -3′ for OAS1p42-CTIL) and the respective S*fi*I-fragments were subcloned in place of OAS1p46 in the pLV lentiviral vector plasmids described above. To generate the human OAS1 p46 Δ12 aa and A32 aa C-terminal truncations, the pLV-S*fi*I-OAS1p46 lentiviral vector plasmid was modified by overlap extension PCR (using primer pair 5′-CTC TCG GAT CCG GCC GAG AGG GCC- 3′ and 5′ -TCT CTC GGC CAG AGA GGC CTC ATC AGA GGA TGG TGC ACT GGA GTG TGC TGG G-3′ for OAS1 p46 Δ12 aa, using primer pair 5′-CTC TCG GAT CCG GCC GAG AGG GCC- 3′ and 5′ -TCT CTC GGC CAG AGA GGC CTC AGA GGA TGG TGC ATT TCT GAT ACC TCC TGG GAT CGT- 3′ for OAS1 p46 Δ32 aa) and the respective S*fi*I-fragments were subcloned into the pLV lentiviral vector plasmids described above. The four most frequent OAS1 p46 protein haplotypes as shown on Ensembl were synthesized with flanking S*fi*I sites and subcloned into pLV-EF1a-IRES-Puro-S*fi*I-TagRFP by Genewiz. Lentiviral vectors were produced by transfecting HEK 293T cells as described previously (86) and 0.45 μm-pore-size-filtered supernatant was used to transduce AAT cells (8) and were selected using 2 μg/ml puromycin or 5 μg/ml blasticidin.

Gene editing by CRISPR-Cas9 was achieved using the lentiCRISPRv2-BlastR or lentiCRISPRv2-PuroR one vector system following the established protocols from the Zhang laboratory. CRISPR guides were designed using the CHOPCHOP online tool (https://chopchop.cbu.uib.no). Seven guides and one non-targeting guide per target were sub-cloned into the one vector system between the *Bsm*BI sites using annealed oligonucleotides with directional, compatible *Bsm*BI overhangs and tested for their efficacy to ablate RNase L, OAS3 or OAS1 expression respectively. The following target sequences for the guides were used: non-targeting control guide (‘NTC’: 5′-GTG ACG TAC CGC TGG AGG TA-3′), **RNase L guides** (guide 1: 5′-GCC GAG TTG CTG TGC AAA CG-3′, guide 2: 5′- TTA TCC TCG CAG CGA TTG CG-3′, guide 3: 5′-CTA TAG GAC GCT TCG GAA TG -3′, guide 4: 5′-TAT AGG ACG CTT CGG AAT GT-3′, guide 5: 5′-TAG TCA TCT TCA GCC GCT AT-3′, guide 6: 5′-TTT ATC CTC GCA GCG ATT GC-3′, and guide 7: 5′-GCA ATC GCT GCG AGG ATA AA -3′), **OAS3 guides** (guide 1: 5′-CAT CAA GGA TCT CTG CGC GG- 3′, guide 2: 5′ -TCA AGG ATC TCT GCG CGG CG- 3′, guide 3: 5′ -CTT GGG TTT GAC GCC GGA GC- 3′, guide 4: 5′ -CGT TCC AGG TGG GAT CAG CG- 3′, guide 5: 5′ - CAA GAT CTA CGG ATG TCA GG- 3′, guide 6: 5′ -AAT TCC AGG GCA TAG ACC GG- 3′, guide 7: 5′ -GAC AGT TTT CAG CAC CCG CG- 3′), **OAS1 guides** (guide 1: 5′ -TCA TCC GCC TAG TCA AGC AC- 3′, guide 2: 5′ -CGG TCT ATG CTT GGG AGC GA- 3′, guide 3: 5′ -TGC ATG CGG AAA CAC GTG TC- 3′, guide 4: 5′ -AAG TTT CCT GTA GGG TCC GC- 3′, guide 5: 5′ -GTA CGA AGC TGA GCG CAC GG- 3′, guide 6: 5′ -AAT CTA TGT CAA GCT CAT CG- 3′, guide 7: 5′ -CGA ACA GGT CAG TTG ACT GG- 3′). RNase L and OAS3 guides were transduced into AAT-OAS1-p46 cells and subsequently selected and cultured in medium additionally supplemented with 5 μg/ml blasticidin (Melford Laboratories). OAS1 guides were transduced into HAT cells, selected, and cultured in medium additionally supplemented with 2 μg/ml puromycin (Melford Laboratories).

### Arrayed ISG expression screening

The ISG overexpression libraries and flow cytometry-based screening have been described previously (5, 6, 86, 90). Briefly, two lentiviral vector ISG libraries consisting of 539 human and 444 macaque ISGs were used to transduce A549-Npro-ACE2 cells (1.25x10^4^ cells/well in a 96-well plate seeded the day before) in the presence of polybrene for 48 h (aiming for an average of >90% transduction), allowing ISG expression from an early HIV-1 mRNA and TagRFP expression from an unspliced late HIV-1 mRNA, the latter used as a marker for transduction. Transduced cells were then infected with synSARS-CoV-2-eGFP (11) in the presence of DMEM supplemented with 2% FCS. At 14 h or 40 h post-infection, cells were trypsinised and fixed in 4% formaldehyde. The percentage of transduced cells (TagRFP-positive) and SARS-CoV-2 infected cells (GFP-positive) were determined by flow cytometry using a Guava EasyCyte flow cytometer (Millipore).

A549-ISRE::GFP cells (gift from Prof. Richard E.Randall (91)) were transduced in the presence of polybrene with the ‘miniscreen’ library of selected ISGs from the SARS-CoV-2 screening. 96 h post-transduction the supernatant was harvested to measure toxicity of the expressed ISGs using the CytoTox-Glow kit (Promega) and cells were fixed in 4% formaldehyde to measure ISRE induction (GFP-positive cells) as surrogate for IFN induction.

The screens in Fig 3A followed the general scheme outlined above. Target cells were seeded in 96-well plates (0.1x10^5^ to 0.6x10^5^ cells/well depending on the cell line) either immediately before transduction (suspension cells) or the day before transduction (adherent cells). Cells were transduced with the SCRPSY ISG library as described above. At 48 h post transduction, the transduced cells were split 1:2 (suspension cells), prior to challenge with a reporter-encoding virus (using a dose lower than MOI 1 to achieve 20-50% infection at the time of fixation). The panel of viruses shown in Fig 3A are: AdV = Human mastadenovirus C (Adenovirus 5), PRV = Suid herpesvirus 1 (Pseudorabies), HSV-1 = Human herpesvirus 1, BoHV-1 =Bovine herpesvirus 1, RVFV = Rift Valley fever phlebovirus, SFTSV = Dabie bandavius (Severe fever with thrombocytopenia syndrome virus), BUNV = Bunyamwera orthobunyavirus, BTV = Bluetongue virus, Rotavirus = Simian Rotavirus A/SA11, CHPV = Chandipura vesiculovirus, VSV = Indiana vesiculovirus, SFV = Semliki Forest virus, CHIKV = Chikungunya virus, HIV-1 = Human immunodeficiency virus 1, IAV PR8 = A/Puerto Rico/8/1934 (H1N1), IAV Cal04 = A/California/04-061-MA/2009 (H1N1), IAV Mallard = A/mallard/Netherlands/10-Cam/1999 (H1N1), MeV = Measles Ed-Zag vac, SeV = Murine respirovirus (Sendai virus), PIV5 = Mammalian orthorubulavirus 5 (Parainfluenza virus 5 or simian virus 5), hMPV = human Metapneumovirus, hRSV = human orthopneumovirus (human respiratory syncytial virus), bRSV = Bovine orthopneumovirus (bovine respiratory syncytial virus), PIV3 = human respirovirus 3 (Parainfluenza virus 3), ZIKA = Zika virus; Further details regarding these viruses, cells and timepoints used for screens presented in Fig3A are available from Enlighten (https://doi.org/10.5525/gla.researchdata.1178). ISG screens were single biological experiments which were followed up with confirmatory ‘miniscreens’ executed in two biological replicates with a typical result being presented.

### Virus infections and titrations

SARS-CoV-2 infection assays, plaque assays and well-clearance assays have been described previously (8). Briefly, well-clearance assays quantify transmitted light (Celigo, Nexcelom) through imaging of a stained cell monolayer. CPE-induced clearance of the monolayer transmits more light compared to uninfected or protected monolayers. AAT cells were seeded at 1.25x10^4^ cells/well and HAT cells at 2x10^4^ cells/well and incubated overnight. The following day, cells were pre-treated or not with IFN for 2 h prior to infection with doses specified in the text/figure. Virus inputs were either normalized using plaque assays on VeroE6 cells to 500 PFU per well or virus input was titrated with 3-fold dilutions adding 50 μl/well.

For plaque assays, AAT derivative cells and VeroE6 cells were seeded in 12-well plates at 3x10^5^ cells/well and incubated overnight. The following day, cells were inoculated with 250 μl of 10-fold logarithmic dilutions of virus stocks prepared in serum-free DMEM. After 1-hour virus adsorption at 37°C the wells were overlaid with 0.6% avicel in MEM. After 3 days incubation, plates were submerged in 8% formaldehyde, washed in PBS, and stained with Coomassie blue for plaque visualization. Plaque assays with SARS-CoV, VSV, EMCV and OC43 were performed under identical conditions to SARS-CoV-2, incubating for 2 days (SARS-CoV, VSV and EMCV) or 5 days (OC43). IAV immunostaining of foci was achieved using mouse anti-influenza A virus nucleoprotein monoclonal antibody clone AA5H (BioRad, MCA400) and visualized with goat anti-mouse IgG (H+L)-HRP conjugate (BioRad, 1721011) and TrueBlue Peroxidase Substrate (KPL, 5510-0030), following standard plaque assay protocols as described previously (88).

For titration of GFP-encoding viruses (PIV3-GFP and RSV-GFP), 96-well plates of AAT derivative cells were seeded with 2x10^4^ cells/well the day before. For titration of SARS-CoV-2-ZsGreen on Calu-3 derivative cells, 5x10^4^ cells/well of a 96-well plate were seeded. The next day cells were infected with serial dilutions of virus for 24 hours (PIV3/RSV) or 40 hours (SARS-CoV-2). Following incubation, the cells were trypsinised and the percentage GFP-positive cells was measured via flow cytometry using a Guava EasyCyte cytometer (Millipore). All virus experiments represent a typical result of at least two biological repeats with 4 technical replicates (plaque assay) or 3 technical replicates (well-clearance and titration assays).

### Western blot analyses

For preparation of cell lysates, cells were seeded in 6-well plates with 1x10^6^ cells/well the day before harvest. Cells were washed once with PBS, harvested in SDS sample buffer (12.5% glycerol, 175 mM Tris-HCl [pH 8.5], 2.5% SDS, 70 mM 2-mercaptoethanol, 0.5% bromophenol blue) and then heated for 10 minutes at 70°C and sonicated. Proteins were subsequently separated on NuPage 4% to 12% Bis-Tris polyacrylamide gels and transferred onto nitrocellulose membranes. Blots were probed with either antibodies raised against actin (mouse JLA20 hybridoma; courtesy of the Developmental Studies Hybridoma Bank, University of Iowa), OAS1 (rabbit polyclonal 14955-1-AP, Proteintech), OAS2 (rabbit polyclonal 19279-1-AP, Proteintech), OAS3 (rabbit polyclonal 21915-1-AP, Proteintech), or the rabbit anti-RNase L monoclonal antibody (Cell Signalling Technology, 27281). Thereafter, membranes were probed with species IgG-specific fluorescently labelled secondary antibodies goat anti-rabbit IgG (Thermo Scientific SA5-10036) or goat anti-mouse IgG (Thermo Scientific SA5-10176) and scanned using a LiCor Odyssey scanner.

### Immunofluorescence

Sub-confluent AAT derivative cells seeded on glass coverslips were infected with CVR-GLA-1 at an MOI of 0.5 for 24 hours. Cells were fixed in PBS/8% formaldehyde, permeabilised with PBS/0.2% TX-100, and blocked with PBS/1% BSA. Immunostaining was performed using a rabbit anti-OAS1 monoclonal antibody [clone D1W3A] (Cell Signaling Technology, 14498) and sheep anti-SARS-CoV-2-nsp5 antiserum, (https://mrcppu-covid.bio, described in (8)) or mouse anti-dsRNA monoclonal antibody (J2, Nordic MUBio 10010500). Secondary antibody staining was performed with Alexa Fluor™ 488 Goat anti-Rabbit IgG and Alexa Fluor™ 594 Donkey anti Sheep IgG both at 1:1000 (Invitrogen). Hoechst 33342 was included in the secondary antibody stain at 5 μg/ml. Coverslips were mounted on glass slides (VWR) using ProLong™ Gold antifade mountant (Thermo Fisher Scientific).

Maximum intensity projection (MIP) images of cell monolayers were acquired with an Airyscan Fast detector fitted to a Zeiss LSM880 confocal microscope. The objective lens used was a Plan-Apochromat 63x/1.4 oil DIC M27 (Carl Zeiss) and gain, laser power and pinhole were synchronised across images. MIP images with pixel scaling of 0.04μm x 0.04μm each comprised a Z-stack of 10 individual slices with a total focal depth of 1.435μm. The OAS1-Alexa 488 was excited at 488 nm and detected in the 495-550 nm range, nsp5-Alexa 594 was excited at 594 nm and detected with a long pass 605nm filter, and Hoechst was excited at 405 nm and detected in the 420-480 nm range. Post-acquisition, the contrast of images within each set were optimised using Zen software (Carl Zeiss) to equal degrees for the vector, p42, p42-CTIL and p46 C397A samples, whereas the histogram maximum was increased independently in the p46 sample shown in (Figure 4I) to prevent oversaturation in the green channel due to strong perinuclear concentration. Images were acquired as 8-bit *.czi files and exported as 8-bit TIFF files.

For dsRNA and OAS1 colocalization analysis, single-slice images were acquired as above but with a gallium arsenide phosphide photomultiplier tube (GaAsP-PMT) detector. The pinhole size was adjusted to obtain an equal focal depth of 2 μm in each channel. Images in each channel were acquired sequentially: OAS1-Alexa 488 was excited at 488 nm and detected in the 493-608 nm range, dsRNA-Alexa 594 was excited at 594 nm and detected in the 599-735 nm range and Hoechst was excited at 405 nm and detected in the 426-500 nm range. To quantify colocalization of dsRNA with OAS1, the Colocalization tool in Zen 3.2 software (Blue version, Carl Zeiss) was used to generate a weighted colocalization coefficient for the dsRNA-Alexa 594 channel in infected cells. Each data point represents a distinct Region of Interest encompassing an individual cell. Presented confocal images are from one of at least two independent experiments performed on separate days.

### In situ-hybridisation

Formalin-fixed and paraffin-embedded (FFPE) lung tissue of 2 patients with confirmed SARS-CoV-2 infection (C21-20: 79 years, male; C19-20: 56 years, male) were used. As control tissue, FFPE lung of a healthy, 62-years old, male donor was used (NBP2-30182, Novusbio, cat. No 0028000B). Additionally, FFPE respiratory nasal epithelium (Amsbio, code: AMS-41022-NE) from 3 healthy donors (patient 3 = specimen 100229 (35 years, female); patient 4 = specimen 100235 (31 years, female); patient 5 = specimen 100233 (37 years, female) was used.

For the detection of gene-specific RNA by *in situ*-hybridisation, the RNAscope 2.5 HD Reagent Kit-RED (code: 322350, Advanced Cell Diagnostics) and the probes (code: NCOA7 1029911-C1, ZBTB42 1029921-C1, OAS1 1029931-C1, ANKFY1 1029941-C1, UNC93B1 1029951-C1, SCARB2 1029961-C1; Advanced Cell Diagnostics) were designed (gene bank no: NM_001199622, NM_001137601, NM_016816, NM_016376, NM_030930, NM_005506) and purchased. As positive and negative control, a human ubiquitin and a plant probe were used, respectively (codes: 310041 and 310043, Advanced Cell Diagnostics). The protocol was followed according to the manufacturer’s instructions. This work (ethics approval number 32077020.6.0000.0005) was approved in May 2020 by the National Committee in Ethics and Research, Brazil in COMISSAO NACIONAL DE ETICA EM PESQUISA. Informed consent was obtained from all study participants.

### OAS1 iCLIP2

iCLIP procedures were performed according to iCLIP2 (35), with additional modifications (36). In detail, 3.5x10^6^ AAT-OAS1p46-RNase L CRISPR Guide 5 cells were seeded into 10cm tissue culture plates. The following day, cells were either infected with SARS-CoV-2 CVR-GLA-1 at MOI 2 in a 6 ml volume of DMEM supplemented with 2% FCS or mock treated with medium only. After 1 hour virus/mock adsorption the medium was changed to 12 ml fresh DMEM supplemented with 2% FCS and incubated for 24 hours. Cells were washed once in ice cold PBS before UV irradiation at 254nm with 150 mJ/cm^2^. Cells were lysed in 1 ml iCLIP lysis buffer per plate (50 mM Tris-Cl [pH7.4], 100 mM NaCl, 1% Igepal CA-630, 0.1% SDS, 0.5% Sodium Deoxycholate, 0.2mM AEBSF) for 30 minutes at 4°C. The experiment was performed in three biological replicates per mock and SARS-CoV-2 infected samples, prepared on separate days with two different virus stocks. Per sample, 4 Units TurboDNase (Life Technologies, AM2238) were added and lysate precleared by centrifugation for 10 minutes at 16000 g at 4°C. A further 4 Units Turbo DNase and 10 Units RNase I (Life Technologies, AM2294) per sample were added and incubated for 3 minutes at 37°C at 1100 rpm. 200 U Ribolock (Life Technologies, EO0382) per sample were added and incubated for 3 minutes on ice. Lysates were pre-cleared with 50 μl Protein A/G Sepharose (ThermoFisher Scientific, 20423) for 30 minutes at 4°C with rotation. Supernatants were split and incubated with either 7.5 mg OAS1 Rabbit monoclonal antibody (Clone D1W3A, Cell Signaling Technology, 14498S) or 7.5 mg Rabbit Isotype control (BD Biosciences, 550875) for 45 minutes at 4°C with rotation followed by binding with further 50 μl Protein A/G Sepharose for 45 minutes at 4°C. Beads were washed twice with 1 mL high salt buffer (50 mM Tris-Cl [pH7.4], 1 M NaCl, 1mM EDTA, 1% Igepal CA-630, 0.1% SDS, 0.5% Sodium Deoxycholate, 0.2mM AEBSF), twice with 1 mL of medium salt buffer (50 mM Tris-Cl [pH7.4], 250mM NaCl, 1mM MgCl2, 0.05% Igepal CA-630, 0.2mM AEBSF), and twice with 1 mL of PNK wash buffer (20 mM Tris-HCl [pH 7.4],10 mM MgCl2, 0.2% Tween-20). RNA was dephosphorylated at 37°C for 40 minutes at 1100 rpm in PNK buffer (50 mM Tris-HCl [pH 6.5], 10mM MgCl2, 1mM DTT) with 5 U PNK (NEB, M0201L), 0.25 U FastAP alkaline phosphatase (Thermo Scientific, EF0654), 0.5 U Turbo DNase and 20 U Ribolock. Beads were washed once with PNK wash buffer, twice with high salt buffer and twice with PNK wash buffer. L3-IR-adapter were generated following irCLIP (92) with the sequence /5Phos/AGATCGGAAGAGCGGTTCAGAAAAAAAAAAAA/iAzideN/AAAAAAAAAAAA/3 Bio/ ordered from IDT. The Adapter was ligated using 10 U T4 RNA ligase (Fisher Scientific, 10669690), 20U Ribolock, 4U PNK, 18% PEG 8000 (Sigma-Aldrich, P1458-25ML), 5% DMSO at 16°C 1100 rpm overnight in the dark. Beads were washed once with PNK wash buffer, twice with high salt buffer and twice with PNK wash buffer. Samples and inputs were denatured in 1x NuPage LDS sample buffer (Thermo Fisher, NP0007) with 100 mM DTT at 70°C for 5 minutes and separated on a 4−20% Mini-PROTEAN® TGX™ (Biorad, 4561093). Protein-RNA complexes were transferred onto an iBLOT2 nitrocellulose membrane (Fisher Scientific, IB23001) and visualised using a Licor Odyssey CLx. The regions corresponding to 42-130 kDa were excised and digested using 20 μl Proteinase K (Roche, 3115828001) in 10 mM Tris-HCl pH 7.4, 100 mM NaCl, 1 mM EDTA, 0.2% SDS at 50°C for 60 minutes at 1100 rpm. RNA was purified via acidic (pH 6.6-6.9) Phenol:Chloroform:Isoamyl Alcohol (Sigma, P3803) followed by RNA cleanup using Zymo RNA Clean & Concentrator-5 (ZYMO research, R1013). A size matched input (SMI) control was extracted in parallel and adapter ligation performed. The SMI was treated with 5 U PNK and 0.5 U FastAP and 20 U Ribolock in PNK buffer for 40 minutes at 37°C at 1100 rpm followed by MyONE silane beads (Life technology, 37002D) purification. L3-IR-adapter ligation was performed with 15 U T4 RNA ligase in 1x ligase buffer with 1.5% DMSO, 16% PEG8000 and 0.1 μM L3-IR-adapter for 75 minutes at room temperature followed by MyONE purification. SMI was treated with 25 U 5′Deadenylase (NEB, M0331S) and 15 U RecJf endonuclease (NEB, M0264S) and 20 U Ribolock in 1x NEB buffer 2 with 8% PEG8000 for 1 hour at 30°C then 30 minutes at 37°C at 1100 rpm followed by a MyONE bead purification. RNA from SMI and iCLIP samples were reverse transcribed using Superscript IV (Invitrogen, 18090010). RNA was hydrolysed by addition of 1.25 ul 1 M NaOH and incubation at 85°C for 15 minutes before neutralisation with 1.25 ul 1 M HCl. cDNA was purified using MyONE silane beads (Life technology, 37002D). Adapters unique to each sample (35) were ligated to the cDNA by mixing 2 μl of 10 μM adapter to 5 μl cDNA and 1 μl DMSO, incubating at 75°C for 2 minutes before placing on ice. Ligation was performed by addition of 15 U T4 RNA ligase in 1x RNA ligase buffer with 18% PEG8000 at 20°C overnight at 1100 rpm. Adapter ligated cDNA was purified again with MyONE beads before PCR amplification. Initial amplification was performed using 2x Phusion HF Master mix (NEB, M0531L) with P5Solexa_s and P3Solexa_s for 6 cycles followed by ProNex (Promega, NG2001) size selection. Optimal qPCR cycles were determined on a Quantstudio 3 (ThermoFisher) using EvaGreen Plus (Biotium, 31077-T) and 2x Phusion HF Mastermix and P5/P3 Solexa primer. Final PCR products were purified using 2 rounds of ProNex Size selection. Libraries were quantified using a Qubit 4 Fluorometer (Thermo Fisher Scientific) and a TapeStation (Agilent). Each group of samples were pooled equimolarly and then were mixed at the following proportions: 51.7% OAS1 library pool, 38.7% size matched input library pool and 8.6% negative control IgG. Pooled pool was sequenced on a NextSeq 550 sequencer with a High output cartridge. The raw data are available from GEO under accession number GSE182394.

### iCLIP2 data processing

To identify OAS1 binding sites on human and SARS-CoV-2 RNAs, reads in the raw fastq files from sequencing were demultiplexed to separate samples according to the sample barcodes using Je suite. Sample barcodes and sequencing adapter sequences were trimmed off using cutadapt. Reads were then mapped to a concatenated human (GRCh38, ENSEMBL Release 104) and SARS-CoV-2 (NC_045512.2) genome using STAR with end-to-end alignment mode. The PCR duplicated reads were collapsed to individual reads based on unique molecular identifier (UMI) barcodes using Je suite. The GRCh38 and NC_045512.2 annotations were pre-processed with htseq-clip suite to generate sliding windows (Human 50 nt window, 20 nt step size; SARS-CoV-2: 10 nt window, 2 nt step size) (available at https://htseq-clip.readthedocs.io/en/latest/overview.html). Uniquely aligned reads were then used to extract crosslink truncation site (position-1 relative to the 5′-end of the read start) using bedtools and htseq-clip/extract and quantified using htseq-clip/count. For peak-calling, we used the R/Bioconductor DEWSeq package to identify significantly enriched sliding windows in OAS1 immunoprecipitated samples over the corresponding size-matched input control samples (adjusted p-value < 0.01, log2 fold change > 2). IHW R package was used for multiple hypothesis correction. To remove background signal resulting from non-specific binding of RNA in our immunoprecipitation experiments, sliding windows harboring more crosslink counts (reads per million, log2 fold change > 2) in IgG samples compared to OAS1 immunoprecipitation samples were removed from further analysis. Overlapping significant windows were merged to binding regions, and these sites were curated to 6 nt long binding peaks based on peak width and maxima using PureCLIP post-processing R scripts. Binding regions were overlapped with GRCh38 gene annotation (ENSEMBL Release 104) using GenomicRanges R package. Small non-conding RNA assignment was given priority unless the binding site spanned annotated RNA junctions.

To assess the main driver of differences in the iCLIP samples, we performed a principal component analysis (PCA). First, we performed library size correction and variance stabilisation transformation implemented in DESeq2. We then used the 1000 sliding windows showing the highest variance to perform PCA using the prcomp function implemented in the base R. For quantification of base composition around the start of the 5′-UTR sequence, reads mapping to the 5′-UTR were extracted using BBMap (http://jgi.doe.gov/data-and-tools/bb-tools/) with kmer=25 mode. Then the 5′-most base of unique reads was extracted from aligned reads and the composition of bases at the 5′start site and the 5′ off-template positions were quantified using bam-readcount (https://github.com/genome/bam-readcount). To assess enrichment of iCLIP reads mapping to each chromosome contigs, Samtools/idxstat was used to report alignment summary statistics of deduplicated reads. The ratio of observed-to-expected read count was calculated using samtools module of MultiQC software.

To identify enriched motifs within iCLIP hits, 25 nucleotides either side of the peak in signal for each hit was taken as the input. Gene and region-matched sequences were used as background for ungapped motif prediction. Ungapped motif prediction was performed using both MEME (40) and HOMER (41) software and the top predicted motif was selected for each. Gapped motif prediction was performed using GLAM2 (40), either allowing for reverse strand search, or not. The top predicted motif was selected for each of these.

### Synteny analysis

The Ensembl web database was used for assessing the OAS1 locus genome synteny between the human genome (GRCh38.p13) and the available Rhinolophus species, *R. ferrumequinum*, genome (mRhiFer1_v1.p). The syntenic region between the human OAS1 exon 7 (ENSE00003913305) and the horseshoe bat genome was examined to identify a region in the latter genome sequence starting at position 7,833,728 of scaffold 25 of the mRhiFer1_v1.p primary assembly that lacked synteny to the human genome. Incidentally, the non-syntenic region started in-frame where the p46 ‘CTIL’ encoding human sequence would have been. We extracted the 580bp *R. ferrumequinum* sequence span up to where synteny resumes to the human genome and used hmmscan (HMMER 3.2.1) (93) to search against the Dfam database (94) for transposable elements present in the sequence. Two confident matches were identified, one to a partial MER74A-like LTR element at the very start of the non-syntenic sequence and one to a L1-like retrotransposon element at the 3′ end of the sequence. The data and code used for this method are publicly available at GitHub repository https://github.com/spyros-lytras/bat_OAS1 (95).

### In silico genome screening

To explore how far back in time this LTR insertion at the OAS1 locus took place we used the Database-integrated genome-screening (DIGS) software (96). DIGS uses a nucleotide or amino acid sequence probe to perform a BLAST similarity search through genome assemblies. We collected a set of 44 Chiroptera species genome assemblies to perform three in-silico screens.

We first used the nucleotide sequence of the syntenic region of *R. ferrumequinum* to human exon 7 (Ensembl) and the adjacent 580bp region with the detected LTR insertion until homology resumes to the human genome as a probe. The DIGS screen was conducted using a minimum blastn bitscore of 30 and minimum sequence length of 30 nucleotides. Matches were aligned using MAFFT v7.453 and inspected for covering all regions of the probe. The second screen used the CAAX terminal amino acid sequence homologous to that encoded by the human exon 7 of 5 previously annotated bat OAS1 proteins holding a CAAX terminus. The minimum tblastn bitscore was set to 60 and minimum sequence length to 40 nucleotides. The translated sequences of hits were aligned and only hits with a CAAX domain present were retained. Finally, to cross-validate that sequence hits of the CAAX domain search are part of the OAS1 locus, we performed a screen using the *R. ferrumequinum* OAS1 C-terminal domain amino acid sequence as a probe with a minimum tblastn bitscore of 100 and minimum sequence length of 100 nucleotides. Only hits of the CAAX search found on scaffolds with a detected OAS1 locus in the third search were maintained in the analysis. It is worth noting that lack of an OAS1 domain detected on the same scaffold as hits in the CAAX sequence search is most likely a result of low genome assembly quality. Regardless the hits were excluded for completeness. The data and code used for this method are publicly available at GitHub repository https://github.com/spyros-lytras/bat_OAS1 (95).

### PDE analysis

To examine the diversity of PDE proteins encoded by coronaviruses, we first constructed an HMMER protein profile. Two seemingly independently acquired PDEs are encoded by the NS2 of Embecoviruses (62) and NS4b of MERS-like coronaviruses (62) respectively. Group A rotavirus (RVA) has also been described to encode a protein with a homologous PDE domain and similar biological function (70). Finally, the AKAP7 mammalian protein holds a PDE domain that has been experimentally shown to complement the function of murine coronaviruses’ NS2 activity (69). We aligned the amino acid sequence of the PDE domains of the OC43 NS2 (AAT84352.1), the MERS NS4b (AIA22866.1) and the NS4b proteins of 2 more bat Merbecoviruses HKU5 (YP_001039965.1) and SC2013 (AHY61340.1), the AKAP7 proteins of Rattus norvegicus (NP_001001801.1), Mus musculus (NP_001366167.1) and humans (NP_057461.2) (as their homology to CoV PDEs has been previously characterized) and the Rotavirus A VP3 protein (AKD32168.1). The alignment was then manually curated using Bioedit based on the homology described in the literature. The final alignment was used to produce an HMM profile using the HMMER suite (v3.2.1) (93).

All complete Coronaviridae sequences were downloaded from the NCBI Virus online database (97). Only sequences with an annotated host and length above 25,000 bp were retained and viruses of “severe acute respiratory syndrome-related coronavirus” species with a human host were excluded, producing a dataset of 2042 complete or near-complete coronavirus genomes. The EMBOSS getorf program was used to extract the translated sequences of all methionine starting open reading frames (ORFs) with length above 100 nucleotides from the filtered virus genome dataset. All putative ORFs were then screened against our custom PDE HMM profile using hmmscan (93). The data and code used for this method are publicly available at GitHub repository https://github.com/spyros-lytras/bat_OAS1 (95).

### ISG expression in gastrointestinal and respiratory tissue and an interferome database

The 6 key genes (UNC93B1, SCARB2, ANKFY1, NCOA7, ZBTB42, OAS1) that were hits in our screens, SARS-CoV-2 cofactors (ACE2, TMPRSS2, CSTL) and RNase L were examined for their gene expression across respiratory and gastrointestinal tissues using 17,382 RNA-sequencing datasets from the Genotype-Tissue Expression (GTEx) v8 database (GTExConsortium, 2020). Gene expression is shown as log10 transform of transcripts per million (TPM). The respiratory tissue here includes Lung and Minor Salivary Gland tissues, while gastrointestinal tissue includes Colon, Esophagus, and Small Intestine tissues. To visualize the IFN responsivity in other datasets, an interferome database was used. Data from the Interferome v2.01 (21) was downloaded (http://www.interferome.org/). The database was searched for each candidate effector and the following additional search criteria were used: Interferon Type I, Species *Homo Sapiens*, Fold Change Up/Down 1.0. The retrieved experimental data of those genes was downloaded as a text file and used for downstream analysis.

### MAIC analysis

The background dataset in MAIC analysis was created as described previously (10). MAIC was then run with the human and macaque lists, each independently, to determine the overlap between these lists and the manually curated systematic review of host factors associated with betacoronavirus literature.

### Clinical data analysis

499 whole-blood patient transcriptomes with known disease outcomes were obtained from the ISARIC4C consortium (https://isaric4c.net/). Ethical approval was given by the South Central-Oxford C Research Ethics Committee in England (reference 13/SC/0149), and by the Scotland A Research Ethics Committee (reference 20/SS/0028). The study was registered at https://www.isrctn.com/ISRCTN66726260. Informed consent was obtained from all study participants https://isaric4c.net/protocols/. Underlying data relating to Fig. 5 can be accessed via Edinburgh DataShare (https://doi.org/10.7488/ds/3139">);

Pre-processed and STAR (98) mapped paired-end reads of 499 whole-blood patient transcriptomes with known disease outcomes from the ISARIC4C study were analysed to stratify mild (hospitalized but not ICU-admitted patients) and severe (ICU admitted and/or deceased) patients further into p46 +ve and p46 -ve groups. Using alignment files as input, strand-specific splice-junction-level counts for each sample were generated using QoRTs (Quality of RNA-seq Tool-Set) (99). QoRTs generates a set of non-overlapping transcript features from the genome annotation, assigns a unique identifier to each feature, and generates counts for each annotated transcript subunit. To validate the method, we applied this method to RNA-seq data from IFN treated A549 cells, which have the AA genotype at Rs10774671 (29). We used the samples A549Cas9Clone1, 3, 4, 7 and 10 that were mock treated (NO IFN) or treated with IFN (IFNbeta) using data retrieved from the European Bioinformatics Institute (EBI) under project accession number PRJEB29677. Based on the presence or absence of p46 junction counts, mild and severe patient samples were sub-divided further into p46 +ve (mild and severe) and p46 -ve (mild and severe) groups. JunctionSeq (100) was used to perform differential usage analysis of both exons and splice junctions, using a design model specifying sample/group types. Differential usage results of p46 junction (named J080 by the QoRTs) and the region of exon 5 encoding the C-terminal end of p42 (VRPPASSLPFIPAPLHEA) (named E037 by the QoRTs) were interpreted and visualized using functions of JunctionSeq.

Disease severity and survival were compared in patients with and without expression of the p46 transcript. Severe disease was classified as ‘ICU admission’ or death, and mild disease as ‘no ICU admission’ and alive at discharge from hospital. Survival was classified as death or ‘alive at discharge from hospital’. Binary logistic regression was used to estimate odds ratios (ORs) and 95% confidence intervals (CIs) with and without adjustment for the effects of age, sex and ethnicity. Analyses were implemented using IBM SPSS Statistics version 25 (IBM Corp. Armonk, USA). To detect whether one patient group stochastically expressed more of an OAS1 isoform than the other group, we used the non-parametric Mann-Whitney U test comparing patients classified as experiencing mild or severe COVID-19. Similarly, where multiple groups were compared, (right hand panel of Fig. 4E) we used the non-parametric Kruskal-Wallis rank sum test followed by post-hoc analysis using the Dunn test. The G frequencies in different populations (at Rs10774671) were extracted from the 1000 genomes project via ensembl. The populations considered were AFR (Africa), AMR (American), EAS (East Asian), EUR (European) and SAS (South Asian). These populations were further subdivided into ASW (African ancestry in SW USA), ACB (African Caribbean in Barbados), BEB (Bengali in Bangladesh), GBR (British from England and Scotland), CDX (Chinese Dai in Xishuangbanna, China), CLM (Colombian in Medellin, Colombia), ESN (Esan in Nigeria), FIN (Finnish in Finland), GWD (Gambian in Western Division − Mandinka), GIH (Gujarati Indians in Houston, Texas, United States), CHB (Han Chinese in Beijing, China), CHS (Han Chinese South, China), IBS (Iberian populations in Spain), ITU (Indian Telugu in the U.K.) JPT, (Japanese in Tokyo, Japan), KHV (Kinh in Ho Chi Minh City, Vietnam), LWK (Luhya in Webuye, Kenya), MSL (Mende in Sierra Leone), MXL (Mexican Ancestry in Los Angeles CA United States), PEL (Peruvian in Lima, Peru), PUR (Puerto Rican in Puerto Rico), PJL (Punjabi in Lahore, Pakistan), STU (Sri Lankan Tamil in the UK), TSI (Toscani in Italia), YRI (Yoruba in Ibadan, Nigeria) and CEU (Utah residents with Northern and Western European ancestry from the CEPH collection).

## Supplementary Material

Supplementary Information

## Data Availability

The raw iCLIP sequencing data are available from GEO under accession number GSE182394. The data and code used for genome analysis are publicly available at GitHub repository https://github.com/spyros-lytras/bat_OAS1 and permanently on Zenodo (95). The ISARIC WHO CCP-UK study protocol is available at https://isaric4c.net/protocols; study registry https://www.isrctn.com/ISRCTN66726260. Underlying data relating to Fig. 5 can be accessed via Edinburgh DataShare (https://doi.org/10.7488/ds/3139); All other underlying data are available from Enlighten (https://doi.org/10.5525/gla.researchdata.1178).

## One-Sentence Summary

A common genetic polymorphism determines whether people make a membrane-associated form of OAS1 that inhibits SARS-CoV-2.
