# Supplementary material for: A prenylated dsRNA sensor protects against severe COVID-19
Source: Science. 2021 Oct 29;374(6567):eabj3624. doi: 10.1126/science.abj3624 (PMC7612834; doi:10.1126/science.abj3624)
Supplement: Supplementary file 2 — Figs. S1 to S7 Table S1 ISARIC4C Author List [file science.abj3624_sm.pdf]

Science AAAS

Supplementary Materials for

A prenylated dsRNA sensor protects against severe COVID-19

Arthur Wickenhagen *et al.*

Corresponding author: Sam J. Wilson, sam.wilson@glasgow.ac.uk

Science **374**, eabj36240 (2021)
DOI: 10.1126/science.abj3624

The PDF file includes:

Figs. S1 to S7
Table S1
ISARIC4C Author List

Other Supplementary Material for this manuscript includes the following:

MDAR Reproducibility Checklist

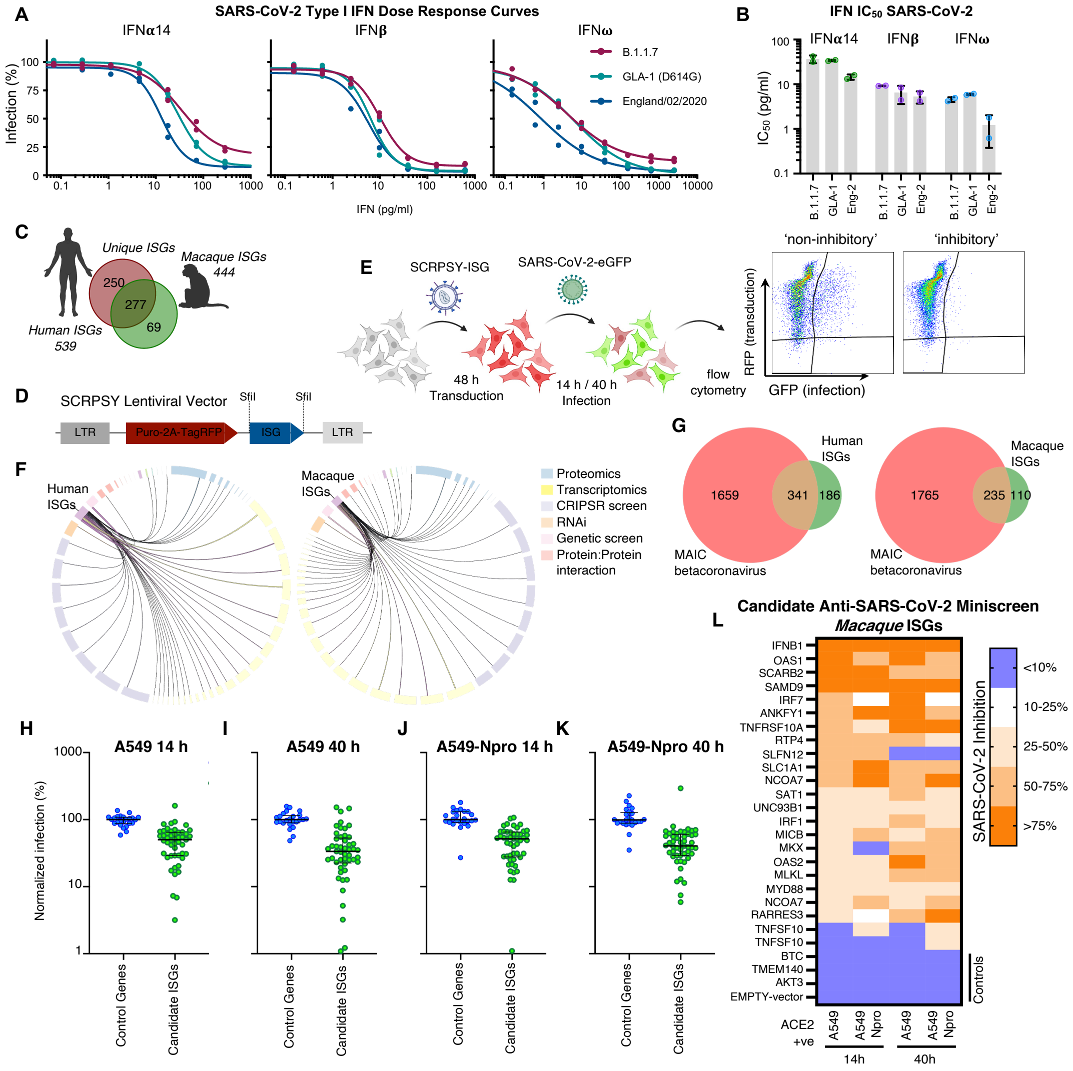

Fig. S1. Arrayed ISG expression screening schematic and extended data.

(A) Dose response curves evaluating the ability of type I IFN pretreatment to protect A549-ACE2-TMPRSS2 (AAT) cells from SARS-CoV-2 infection (B.1.1.7= B.1.1.7 isolate '212', GLA-1 (D614G)= CVR-GLA-1 and England/02/2020= England/02/2020). Normalized infection (% well clearance quantified by transmitted light) **(B)** IC_{50} estimates derived from A. **(C)** A diagrammatic representation of the ISG libraries used. **(D)** A diagrammatic representation of the SCRPSY lentiviral vector used in Figure 1A-D. **(E)** Schematic diagram of the ISG screening method used in Figure 1A and confirmatory 'miniscreens' (created using biorender.com). **(F)** Circos diagram illustrating the overlap between ISG candidates and datasets curated from betacoronavirus literature. ISG candidates are well supported with evidence from many experimental types (see legend). **(G)** Venn diagram of overlap between the human ISG candidates (left) and macaque ISG candidates (right) with the top 2000 hits from the betacoronavirus MAIC analysis (release:

25/11/2020). **(H-K)** Scatter plots of the individual miniscreens represented in (L) and Figure 1B on A549 (H-I) or A549-Npro (J-K) at 14 or 40 hpi. **(L)** Miniscreen of the ability of macaque candidate effectors identified in Figure 1A alongside controls, to inhibit SARS-CoV-2 in A549 and A549-Npro at 14 and 40 hpi (the equivalent panel for human ISGs presented in Figure 1B).

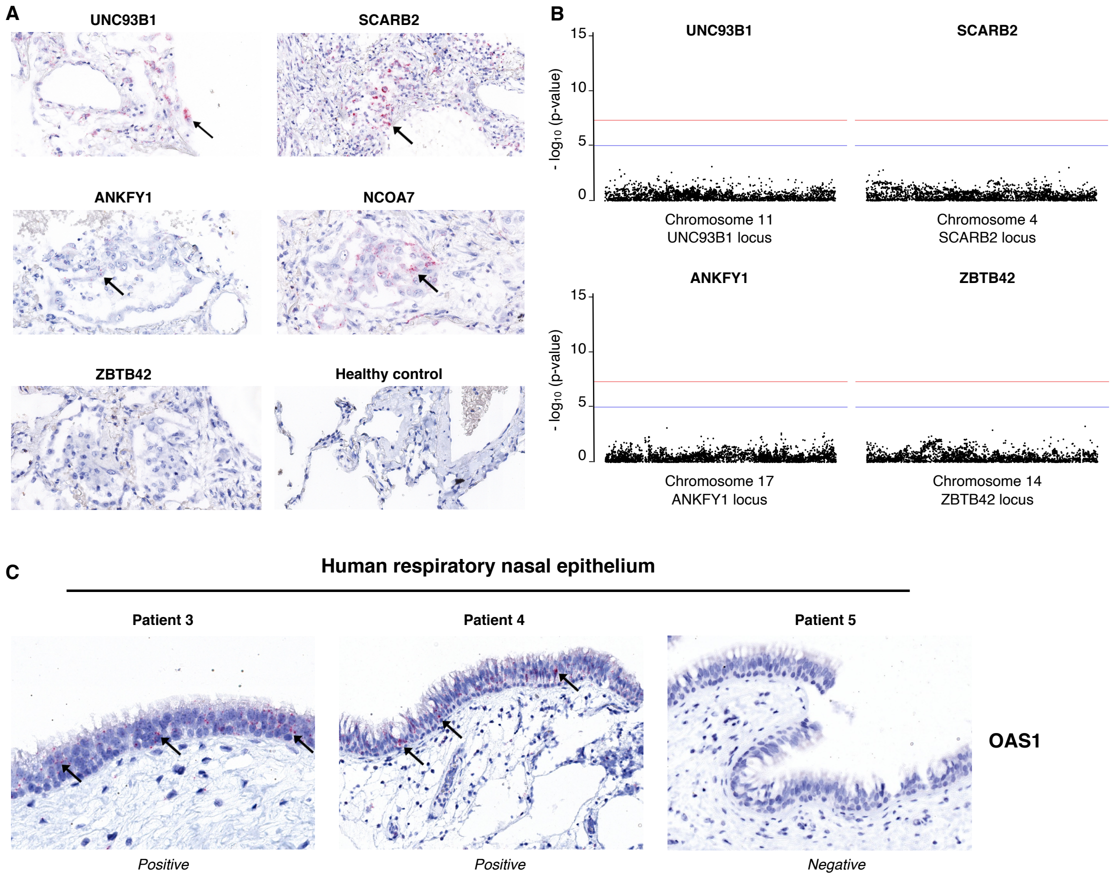

Fig. S2. OAS1 potently inhibits SARS-CoV-2.

(A) Detection of UNC93B1, SCARB2, ANKFY1, NCOA7, ZBTB42 gene expression using RNAscope in FFPE lung tissue of deceased COVID-19 patients compared to healthy control lung tissue. No signal detected for ZBTB42 while gene expression could be detected for the other genes. Arrows indicate staining +ve cells. **(B)** Meta-analysis of the COVID-19 Host Genetics Initiative (covid19hg.org) for genetic variation between critically ill COVID-19 patients and control populations at the gene locus of the UNC93B1, SCARB2, ANKFY1 and ZBTB42 genes with the red line indicating the threshold for significant SNPs (yellow dots). **(C)** Detection of OAS1 gene expression using RNAscope in FFPE nasal epithelium samples from 3 different healthy donors. Gene expression detected or not as indicated below pictures, and arrows show staining +ve cells.

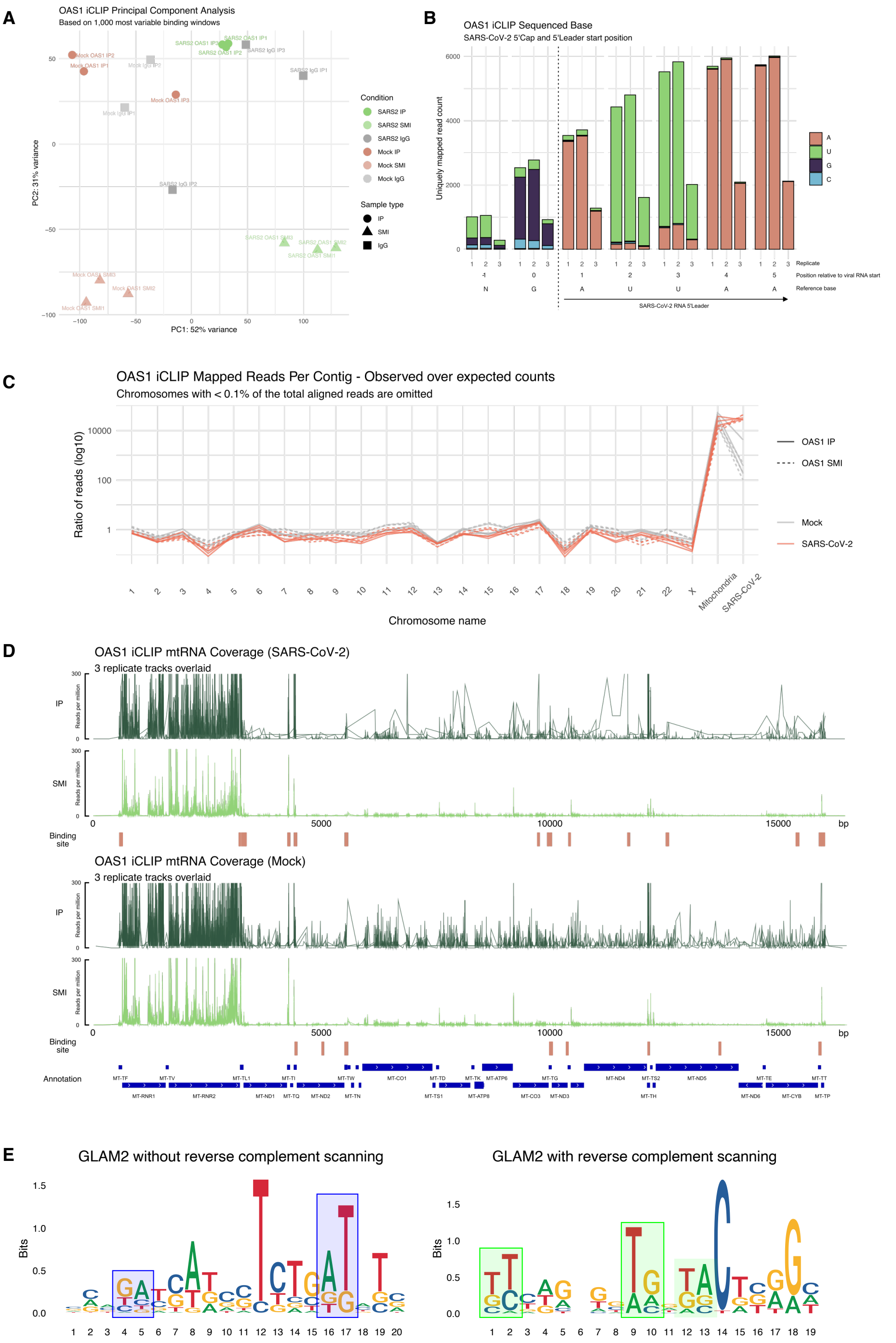

Fig. S3. OAS1 iCLIP2 extended data

(A) Principal component analysis of OAS1 iCLIP2 samples between SARS-CoV-2 and mock infected samples. **(B)** Sequenced base with single-nucleotide resolution at the 5′Cap and the 5′-UTR of SARS-CoV-2. Dotted vertical line indicates SARS-CoV-2 template start

position. **(C)** Proportion of reads uniquely aligned to the host chromosomes, mitochondria, or SARS-CoV-2 genome represented as observed over expected read count ratios. **(D)** iCLIP2 analysis of OAS1 binding sites on mitochondrial RNA. Coverage of 3 replicate tracks overlaid mapped to the mitochondrial genes in the OAS1-IP and a size matched input control (SMI). Both shown for SARS-CoV-2 infected and mock treated samples with OAS1 binding sites shown in red and mitochondrial gene annotation in blue. Y-axis were thresholded at 300 reads pe million (RPM) to clearly show coverage shape differences between IP and SMI samples, otherwise masked by strong non-specific signals. **(E)** Motif prediction of OAS1 binding sites in cellular transcripts using GLAM2 with or without reverse complement scanning.

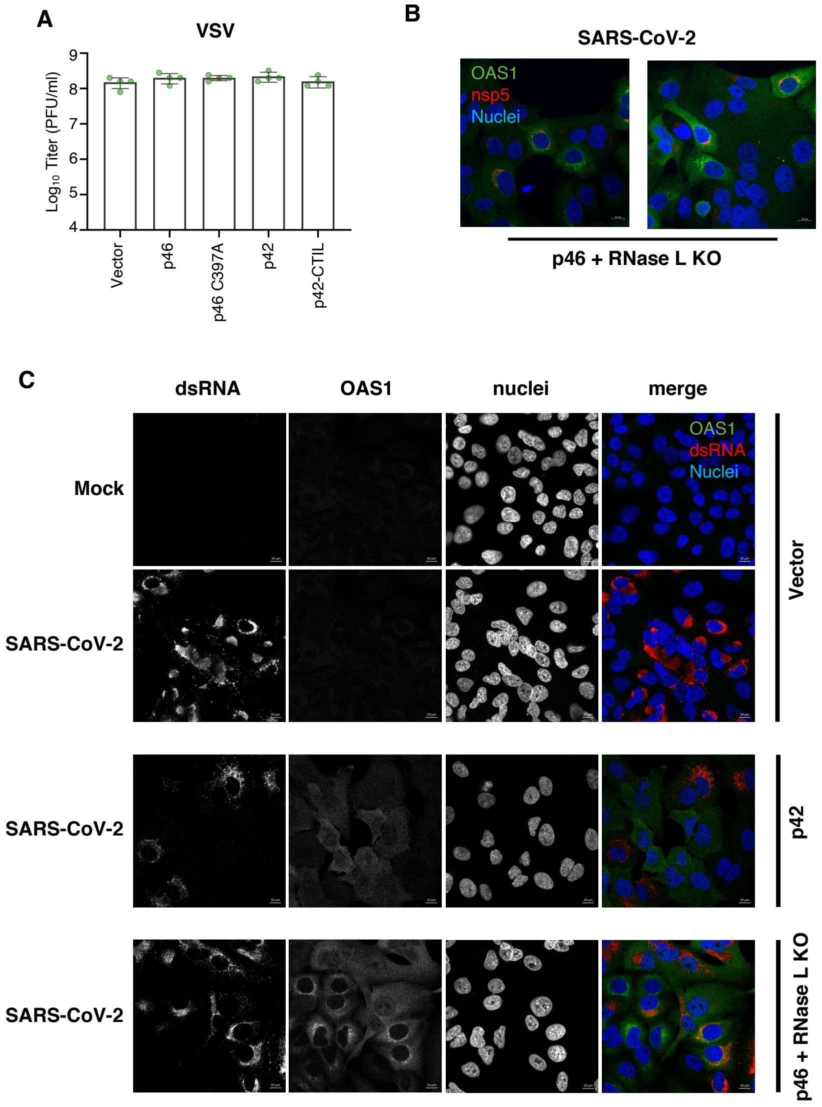

Fig. S4. Extended data OAS1 isoforms

(A) Indiana vesiculovirus (VSV) titer determined by plaque assay in cells modified to express exogenous OAS1 p46, p46-C397A, p42 and p42-CTIL. **(B)** Representative immunofluorescence images of AAT cells expressing OAS1 p46 and RNase L KO infected with SARS-CoV-2. Cells were infected with SARS-CoV-2 isolate CVR-GLA-1 at MOI 0.5 for 24 hours followed by staining with anti-OAS1 (green) and anti-SARS-CoV-2-nsp5 (red) antibodies and nuclear Hoechst stain (blue). **(C)** Immunofluorescence monochrome images of mock or SARS-CoV-2 infected cells relating to Fig 4K. AAT-Vector control, AAT-OAS1p42 and AAT-OAS1p46-RNase L KO cells were mock or SARS-CoV-2 CVR-GLA-1 infected for 24 hours followed by staining with anti-OAS1 (green) and anti-dsRNA (red) antibodies and nuclear Hoechst stain (blue).

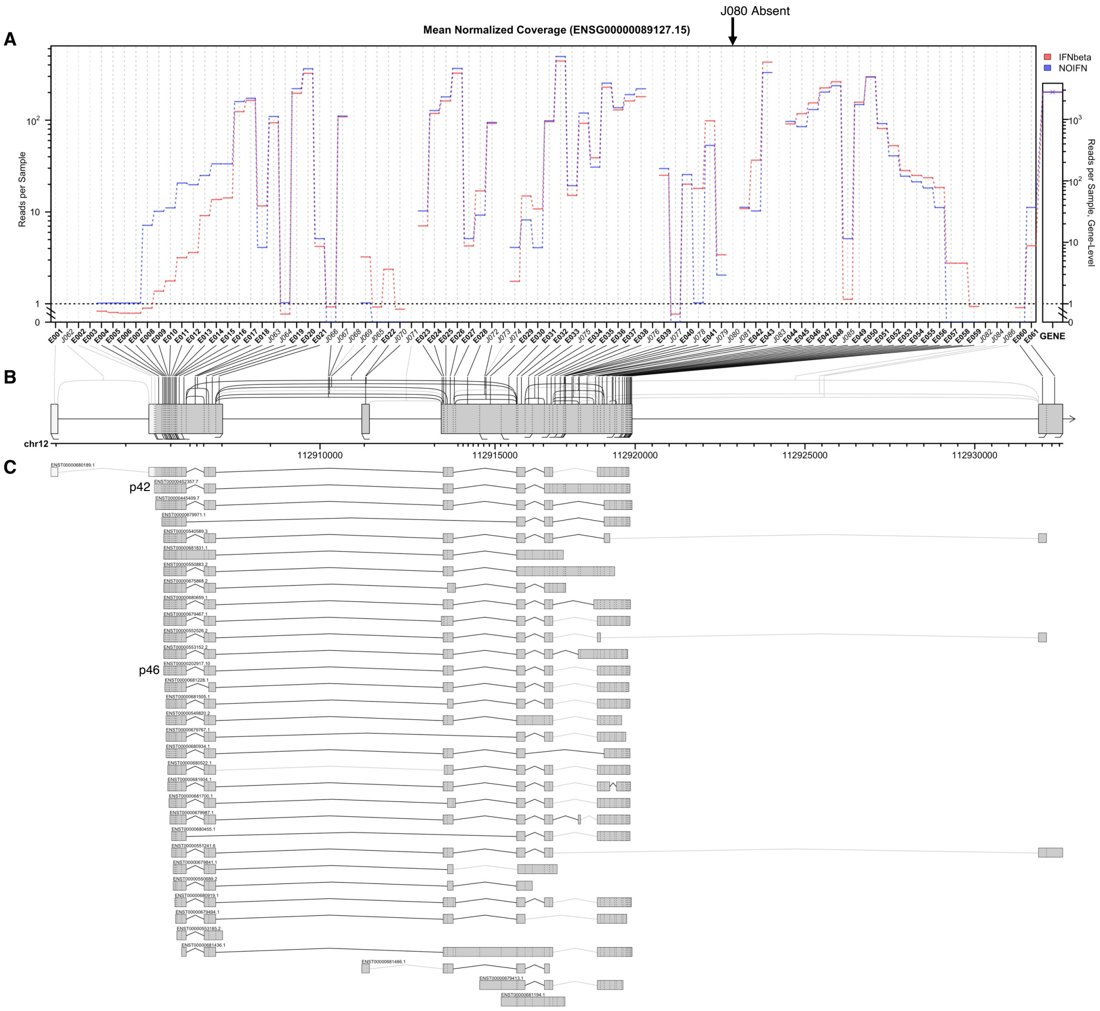

Fig. S5. Transcripts encoding p46 were not detected using JunctionSeq differential usage analysis of OAS1 transcripts from AA homozygous A549 cells.

(A) The expression levels at each junction and exon for every annotated OAS1 transcript (Ensembl) from A549 cells stimulated with IFN (IFNbeta – red line) or not treated (NOIFN – blue line). Junction 80 (J080) is highlighted with an arrow. **(B)** A diagrammatic representation of introns and exons on chromosome 12, highlighting the corresponding chromosomal location to the transcript junctions. **(C)** A diagrammatic representation of the differential exon usage of all known OAS1 transcripts (Ensembl). Splice junctions detected in the RNA-seq data are linked by black lines whereas undetected junctions are linked by light gray lines. The transcripts encoding p46 and p42 proteins are highlighted.

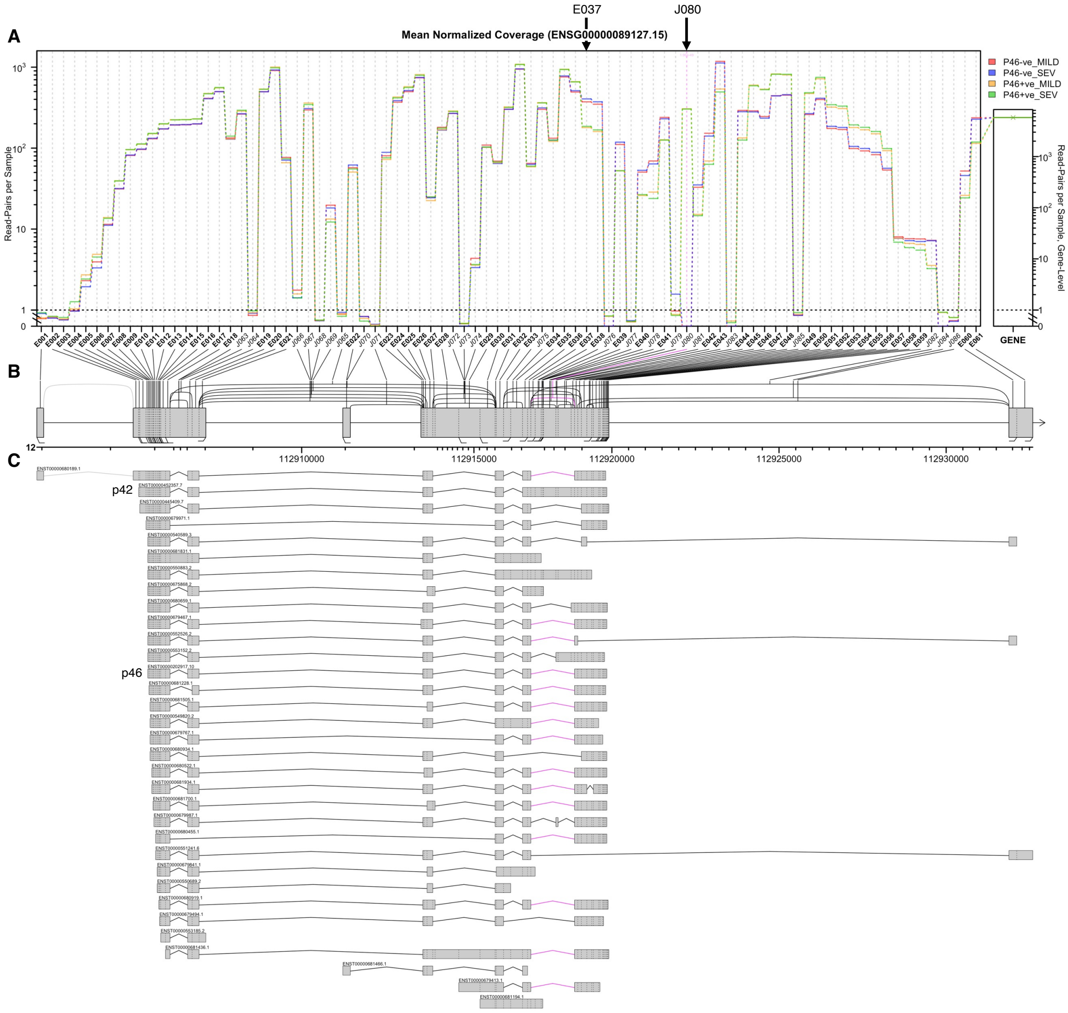

Fig. S6. Strand-specific exon and known splice-junction loci counts of OAS1 transcripts generated by QoRTs from 499 samples were used to stratify patients into p46 transcript positive and negative patients and subjected to differential usage analysis via JunctionSeq.

(A) The expression level at each junction and exon for every annotated OAS1 transcript (Ensembl) from 499 COVID-19 patients. The mild and severe patient groups were subdivided based on presence or absence of junction 80. Patients with no mapped reads were classified as p46 –ve and patients with mapped reads as p46 +ve. Junction 80 (J080) is highlighted with an arrow. **(B)** A diagrammatic representation of introns and exons on chromosome 12, highlighting the corresponding chromosomal location to the transcript junctions. **(C)** A diagrammatic representation of the differential exon usage of all known OAS1 transcripts (Ensembl). Splice junctions detected in the RNA-seq data are linked by black lines whereas absent junctions are linked by light gray lines. The transcripts encoding p46 and p42 proteins are highlighted. Junctions significantly different between groups are highlighted in purple.

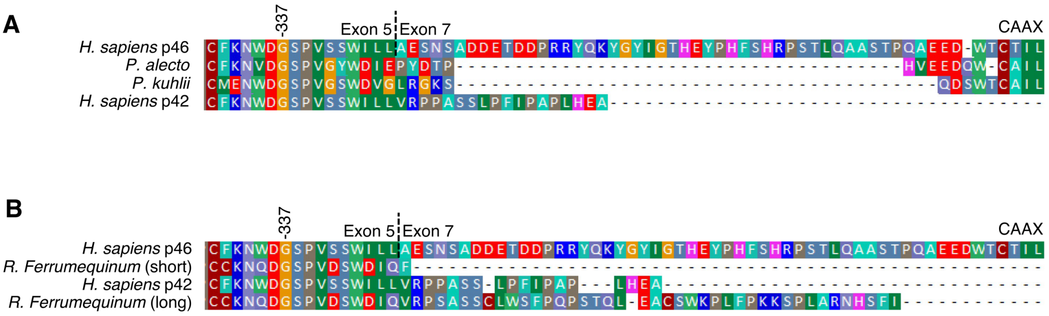

Fig. S7. Alignments of the C-termini of species variants of OAS1.

(A) The C-termini of human p46 (NM_016816) and p42 (NM_002534) are shown aligned with OAS1 from *P. alecto* (NM_001290162) and *P. kuhlii* (XM_036409709). **(B)** The C-termini of human p46 (NM_016816) and p42 (NM_002534) are shown aligned with the short isoform of *R. ferrumequinum* OAS1 (XP_032953023) and the longer *R. ferrumequinum* isoform (ENSRFEP00010015335). Residue 337 (Fig. 4.) and the splice junction specifying the prenylated C-terminus of p46 are indicated.

Table S1.

OAS1 binding sites in viral transcripts identified using iCLIP2.

Reference	Begins	Ends	Strand	Length	Region	IP RPM[a]	SMI RPM[b]	Fold Enrichment	*p* value[c]
NC_045512.2	0	54	+	54	5' UTR	47149.6	11182.3	4.22	1.20E-05
NC_045512.2	1807	1825	+	18	nsp2	128.9	32.3	3.99	8.38E-08
NC_045512.2	26524	26536	+	12	M	257.8	66.8	3.86	2.01E-03
NC_045512.2	22776	22788	+	12	S	126.6	38.7	3.27	1.04E-04
NC_045512.2	24846	24856	+	10	S	185.1	59.2	3.12	5.88E-05
NC_045512.2	26132	26154	+	22	ORF3a	126.6	44	2.88	1.60E-03
NC_045512.2	707	717	+	10	nsp1	205.4	73.6	2.79	4.01E-03
NC_045512.2	1065	1075	+	10	nsp2	87.7	37.5	2.34	9.75E-03
NC_045512.2	2349	2359	+	10	nsp2	88	37.6	2.34	3.48E-03
NC_045512.2	1791	1801	+	10	nsp2	77.9	34	2.29	6.40E-04
NC_045512.2	8933	8943	+	10	nsp4	29.3	24.6	1.19	9.63E-03

[a]The median number of reads mapped per million reads in the OAS1 p46 immunoprecipitation. [b]The median number of reads mapped per million reads in the size matched input control. [c]Significance was determined using independent hypothesis weighting (IHW) and the mean adjusted p-value of sliding windows constituting each binding region are reported. The raw iCLIP sequencing data are available from GEO under accession number GSE182394. Processed iCLIP2 data are available from Enlighten (https://doi.org/10.5525/gla.researchdata.1178).

ISARIC4C Investigators

Consortium Lead Investigator: J Kenneth Baillie.

Chief Investigator: Malcolm G Semple.

Co-Lead Investigator: Peter JM Openshaw.

ISARIC Clinical Coordinator: Gail Carson.

Co-Investigator: Beatrice Alex, Benjamin Bach, Wendy S Barclay, Debby Bogaert, Meera Chand, Graham S Cooke, Annemarie B Docherty, Jake Dunning, Ana da Silva Filipe, Tom Fletcher, Christoper A Green, Ewen M Harrison, Antonia Ying Wai Ho, Peter W Horby, Samreen Ijaz, Saye Khoo, Paul Klenerman, Andrew Law, Wei Shen Lim, Alexander J Mentzer, Laura Merson, Alison M Meynert, Mahdad Noursadeghi, Shona C Moore, Massimo Palmarini , William A Paxton, Georgios Pollakis, Nicholas Price, Andrew Rambaut, David L Robertson, Clark D Russell, Vanessa Sancho-Shimizu, Janet T Scott, Thushan de Silva, Louise Sigfrid, Tom Solomon, Shiranee Sriskandan, David Stuart, Charlotte Summers, Richard S Tedder, Emma C Thomson, AA Roger Thompson, Ryan S Thwaites, Lance CW Turtle, Rishi K Gupta, Carlo Palmieri, Olivia V Swann, Maria Zambon, Marc-Emmanuel Dumas, Julian L Griffin, Zoltan Takats, Kanta Chechi, Petros Andrikopoulos, Anthonia Osagie, Michael Olanipekun, Sonia Liggi, Matthew R Lewis, Gonçalo dos Santos Correia, Caroline J Sands, Panteleimon Takis, Lynn Maslen.

Project Manager: Hayley Hardwick, Chloe Donohue, Fiona Griffiths, Wilna Oosthuyzen.

Project Administrator: Cara Donegan, Rebecca G. Spencer.

Data Analyst: Lisa Norman, Riinu Pius, Thomas M Drake, Cameron J Fairfield, Stephen R Knight, Kenneth A Mclean, Derek Murphy, Catherine A Shaw.

Data and Information System Manager: Jo Dalton, Michelle Girvan, Egle Saviciute, Stephanie Roberts, Janet Harrison, Laura Marsh, Marie Connor, Sophie Halpin, Clare Jackson, Carrol Gamble, Daniel Plotkin, James Lee.

Data Integration and Presentation: Gary Leeming, Andrew Law, Murray Wham, Sara Clohisey, Ross Hendry, James Scott-Brown.

Material Management: William Greenhalf, Victoria Shaw, Sarah E McDonald.

Patient Engagement: Seán Keating.

Outbreak Laboratory Staff and Volunteers: Katie A. Ahmed, Jane A Armstrong, Milton Ashworth, Innocent G Asiimwe, Siddharth Bakshi, Samantha L Barlow, Laura Booth, Benjamin Brennan, Katie Bullock, Benjamin WA Catterall, Jordan J Clark, Emily A Clarke, Sarah Cole, Louise Cooper, Helen Cox, Christopher Davis, Oslem Dincarslan, Chris Dunn, Philip Dyer, Angela Elliott, Anthony Evans, Lorna Finch, Lewis WS Fisher, Terry Foster, Isabel Garcia-Dorival, William Greenhalf, Philip Gunning, Catherine Hartley, Rebecca L Jensen, Christopher B Jones, Trevor R Jones, Shadia Khandaker, Katharine King, Robyn T. Kiy, Chrysa Koukorava, Annette Lake, Suzannah Lant, Diane Latawiec, Lara Lavelle-Langham, Daniella Lefteri, Lauren Lett, Lucia A Livoti, Maria Mancini, Sarah McDonald, Laurence McEvoy, John McLauchlan, Soeren Metelmann, Nahida S Miah, Joanna Middleton, Joyce Mitchell, Shona C Moore, Ellen G Murphy, Rebekah Penrice-Randal, Jack Pilgrim, Tessa Prince, Will Reynolds, P. Matthew Ridley,

Debby Sales, Victoria E Shaw, Rebecca K Shears, Benjamin Small, Krishanthi S Subramaniam, Agnieska Szemiel, Aislynn Taggart, Jolanta Tanianis-Hughes, Jordan Thomas, Erwan Trochu, Libby van Tonder, Eve Wilcock, J. Eunice Zhang, Lisa Flaherty, Nicole Maziere, Emily Cass, Alejandra Doce Carracedo, Nicola Carlucci , Anthony Holmes, Hannah Massey.

Edinburgh Laboratory Staff and Volunteers: Lee Murphy, Nicola Wrobel, Sarah McCafferty, Kirstie Morrice, Alan MacLean.

Local Principal Investigators: Kayode Adeniji, Daniel Agranoff, Ken Agwuh, Dhiraj Ail, Erin L. Aldera, Ana Alegria, Sam Allen, Brian Angus, Abdul Ashish, Dougal Atkinson, Shahedal Bari, Gavin Barlow, Stella Barnass, Nicholas Barrett, Christopher Bassford, Sneha Basude, David Baxter, Michael Beadsworth, Jolanta Bernatoniene, John Berridge, Colin Berry, Nicola Best, Pieter Bothma, David Chadwick, Robin Brittain-Long, Naomi Bulteel, Tom Burden, Andrew Burtenshaw, Vikki Caruth, David Chadwick, Duncan Chambler, Nigel Chee, Jenny Child, Srikanth Chukkambotla, Tom Clark, Paul Collini, Catherine Cosgrove, Jason Cupitt, Maria-Teresa Cutino-Moguel, Paul Dark, Chris Dawson, Samir Dervisevic, Phil Donnison, Sam Douthwaite, Andrew Drummond, Ingrid DuRand, Ahilanadan Dushianthan, Tristan Dyer, Cariad Evans, Chi Eziefula, Chrisopher Fegan, Adam Finn, Duncan Fullerton, Sanjeev Garg, Sanjeev Garg, Atul Garg, Effrossyni Gkrania-Klotsas, Jo Godden, Arthur Goldsmith, Clive Graham, Elaine Hardy, Stuart Hartshorn, Daniel Harvey, Peter Havalda, Daniel B Hawcutt, Maria Hobrok, Luke Hodgson, Anil Hormis, Michael Jacobs, Susan Jain, Paul Jennings, Agilan Kaliappan, Vidya Kasipandian, Stephen Kegg, Michael Kelsey, Jason Kendall, Caroline Kerrison, Ian Kerslake, Oliver Koch, Gouri Koduri, George Koshy, Shondipon Laha, Steven Laird, Susan Larkin, Tamas Leiner, Patrick Lillie, James Limb, Vanessa Linnett, Jeff Little, Mark Lyttle, Michael MacMahon, Emily MacNaughton, Ravish Mankregod, Huw Masson, Elijah Matovu, Katherine McCullough, Ruth McEwen, Manjula Meda, Gary Mills, Jane Minton, Mariyam Mirfenderesky, Kavya Mohandas, Quen Mok, James Moon, Elinoor Moore, Patrick Morgan, Craig Morris, Katherine Mortimore, Samuel Moses, Mbiye Mpenge, Rohinton Mulla, Michael Murphy, Megan Nagel, Thapas Nagarajan, Mark Nelson, Lillian Norris, Matthew K. O'Shea, Igor Otahal, Marlies Ostermann, Mark Pais, Carlo Palmieri, Selva Panchatsharam, Danai Papakonstantinou, Hassan Paraiso, Brij Patel, Natalie Pattison, Justin Pepperell, Mark Peters, Mandeep Phull, Stefania Pintus, Jagtur Singh Pooni, Tim Planche, Frank Post, David Price, Rachel Prout, Nikolas Rae, Henrik Reschreiter, Tim Reynolds, Neil Richardson, Mark Roberts, Devender Roberts, Alistair Rose, Guy Rousseau, Bobby Ruge, Brendan Ryan, Taranprit Saluja, Matthias L Schmid, Aarti Shah, Prad Shanmuga, Anil Sharma, Anna Shawcross, Jeremy Sizer, Manu Shankar-Hari, Richard Smith, Catherine Snelson, Nick Spittle, Nikki Staines, Tom Stambach, Richard Stewart, Pradeep Subudhi, Tamas Szakmany, Kate Tatham, Jo Thomas, Chris Thompson, Robert Thompson, Ascanio Tridente, Darell Tupper-Carey, Mary Twagira, Nick Vallotton, Rama Vancheeswaran, Lisa Vincent-Smith, Shico Visuvanathan, Alan Vuylsteke, Sam Waddy, Rachel Wake, Andrew Walden, Ingeborg Welters, Tony Whitehouse, Paul Whittaker, Ashley Whittington, Padmasayee Papineni, Meme Wijesinghe, Martin Williams, Lawrence Wilson, Sarah Cole, Stephen Winchester, Martin Wiselka, Adam Wolverson, Daniel G Wootton, Andrew Workman, Bryan Yates, Peter Young.
