# Supplementary material for: A prenylated dsRNA sensor protects against severe COVID-19
Source: Science. 2021 Oct 29;374(6567):eabj3624. doi: 10.1126/science.abj3624 (PMC7612834; doi:10.1126/science.abj3624)
Supplement: Supplementary file 3 — MDAR Reproducibility Checklist [file science.abj3624_mdar_reproducibility_checklist.pdf]

Materials Design Analysis Reporting (MDAR) Checklist for Authors

The MDAR framework establishes a minimum set of requirements in transparent reporting applicable to studies in the life sciences (see Statement of Task: doi:10.31222/osf.io/9sm4x.). The MDAR checklist is a tool for authors, editors and others seeking to adopt the MDAR framework for transparent reporting in manuscripts and other outputs.   Please refer to the MDAR Elaboration Document for additional context for the MDAR framework.

Materials

Antibodies	Yes (indicate where provided: page no/section/legend)	n/a
For commercial reagents, provide supplier name, catalogue number and RRID, if available.	Materials and Methods, Western blot analyses.<br>-mouse JLA20 hybridoma; courtesy of the Developmental Studies Hybridoma Bank, University of Iowa<br>-OAS1 (rabbit polyclonal 14955-1-AP, Proteintech)<br>-OAS2 (rabbit polyclonal 19279-1-AP, Proteintech)<br>-OAS3 (rabbit polyclonal 21915-1-AP, Proteintech)<br>-Rabbit anti-RNase L monoclonal antibody (Cell Signalling Technology, 27281)<br>-goat anti-rabbit IgG (Thermo Scientific SA5-10036) or goat anti-mouse IgG (Thermo Scientific SA5-10176)<br>Immunofluorescence<br>-OAS1 monoclonal antibody [clone D1W3A] (Cell Signaling Technology, 14498)<br>- sheep anti-SARS-CoV-2-nsp5 antiserum, (https://mrcppu-covid.bio)<br>- Alexa Fluor™ 488 Goat anti-Rabbit IgG and Alexa Fluor™ 594 Donkey anti Sheep IgG (Invitrogen)<br>-Mouse anti-dsRNA monoclonal antibody (J2, Nordic MUBio 10010500)	

Cell materials	Yes (indicate where provided: page no/section/legend)	n/a
**Cell lines:** Provide species information, strain. Provide accession number in repository **OR** supplier name, catalog number, clone number, **OR** RRID	Materials and Methods -Cell lines, plasmids and viruses<br>A549-ACE2-TMPRSS2 ('AAT') and VeroE6-ACE2-TMPRSS2 ('VAT') cells available from NIBSC with codes CFAR#101004 and CFAR#101003.<br>A549-NPro cells - gift of Prof. Richard E. Randall<br>HT1080 cells - gift of Prof. Stuart Neil<br>Calu-3 cells - gift of Prof. Paul J Lehner<br>VeroE6 -gift of Prof. Michele Bouloy<br>HEK-293T – lab stock<br>(except VeroE6 and Calu-3, all cell lines validated by STR analysis during the project)<br>ISG Screening supplementary material<br>https://doi.org/10.5525/gla.researchdata.1178	
**Primary cultures:** Provide species, strain, sex of origin, genetic modification status.		n/a

Experimental animals	Yes (indicate where provided: page no/section/legend)	n/a
**Laboratory animals:** Provide species, strain, sex, age, genetic modification status. Provide accession number in repository **OR** supplier name, catalog number, clone number, **OR** RRID		n/a
**Animal observed in or captured from the field:** Provide species, sex and age where possible		n/a
**Model organisms:** Provide Accession number in repository (where relevant) **OR** RRID		n/a

Plants and microbes	Yes (indicate where provided: page no/section/legend)	n/a
**Plants:** provide species and strain, unique accession number if available, and source (including location for collected wild specimens)		n/a

**Microbes:** provide species and strain, unique accession number if available, and source	Materials and Methods -Cell lines, plasmids and viruses<br>-CVR-GLA-1 and SARS-CoV-2-ZsGreen (https://mrcppu-covid.bio/)<br>-SynSARS-CoV-2-eGFP - gift from Prof. Volker Thiel<br>-B.1.1.7 '212' was isolated from a clinical sample (gift from Prof. Wendy Barclay)<br>-Indiana vesiculovirus (VSV) - gift of Dr. Megan Stanifer<br>-Influenza A viruses A/Puerto Rico/8/1934 (H1N1) and A/mallard/Netherlands/10-Cam/1999(H1N1) were rescued from reverse genetics systems (gifts from Prof. Ron Fouchier, and Prof. Laurence Tiley, respectively)<br>-Human respirovirus 3 with GFP (PIV3-GFP) was purchased from ViraTree<br>-Human respiratory syncytial virus expressing GFP (RSV-GFP) -gift from Prof. Peter Collins<br>-Encephalomyocarditis virus (EMCV) -gift from Dr. Connor Bamford<br>-Betacoronavirus OC43 (ATCC VR-1558) was purchased from ATCC<br>-SARS-CoV virus isolate (HKU39849, GenBank: AY278491.2) - gift of Prof. Malik Peiris/supplied by Prof. Bart Haagmans<br>ISG Screening supplementary material<br>https://doi.org/10.5525/gla.researchdata.1178	

**Human research participants**	**Yes (indicate where provided: page no/section/legend)**	**n/a**
Identify authority granting ethics approval (IRB or equivalent committee(s), provide reference number for approval.	Materials and Methods – In situ-hybridisation<br>(ethics approval number 32077020.6.0000.0005) was approved on May 2020 by the National Committee in Ethics and Research, Brazil. in COMISSÃO NACIONAL DE ÉTICA EM PESQUISA<br>-Clinical data analysis<br>Ethical approval was given by the South Central-Oxford C Research Ethics Committee in England (reference 13/SC/0149), and by the Scotland A Research Ethics Committee (reference 20/SS/0028). The study was registered at https://www.isrctn.com/ISRCTN66726260.	
Provide statement confirming informed consent obtained from study participants.	Materials and Methods<br>- Clinical data analysis<br>- In situ-hybridisation	
Report on age and sex for all study participants.	Methods and Materials<br>-In situ-hybridisation<br>Clinical data analysis -isaric4c.net/data	

Design

Study protocol	Yes (indicate where provided: page	n/a
For clinical trials, provide the trial registration number **OR** cite DOI in manuscript.		n/a

Laboratory protocol	Yes (indicate where provided: page	n/a
Provide DOI or other citation details if detailed step-by-step protocols are available.		n/a

Experimental study design (statistics details)	Yes (indicate where provided: page	n/a
State whether and how the following have been done**, or** if they were not carried out.		
Sample size determination		n/a
Randomisation		n/a
Blinding		n/a
Inclusion/exclusion criteria	Main text- Prenylated OAS1 is associated with less severe COVID-19<br>When adjusting for age, sex, and ethnicity, 24 cases were excluded as ethnicity was unknown and 6 cases excluded as age was not recorded. This was not specified in advance. "(unadjusted Odds Ratio (OR)=1.57, 95% CI 1.09, 2.25; following adjustment for age, sex, and ethnicity and exclusion of 30 cases with missing data OR=1.58, 95% CI 1.08, 2.30) (Fig. 5G)"	

Sample definition and in-laboratory replication	Yes (indicate where provided: page	n/a
State number of times the experiment was replicated in laboratory	Materials and Methods sections-<br>-Arrayed ISG expression screening<br>-Virus infections and titrations<br>- Immunofluorescence<br>- iCLIP<br>Fig4 legend	
Define whether data describe technical or biological replicates	Materials and Methods sections-<br>-Arrayed ISG expression screening<br>-Virus infections and titrations<br>- Immunofluorescence<br>- iCLIP<br>-Fig4 legend	

Ethics	Yes (indicate where provided: page	n/a
Studies involving human participants: State details of authority granting ethics approval (IRB or equivalent committee(s), provide reference number for approval.	Materials and Methods – In situ-hybridisation<br>(ethics approval number 32077020.6.0000.0005) was approved on May 2020 by the National Committee in Ethics and Research, Brazil. in COMISSÃO NACIONAL DE ÉTICA EM PESQUISA<br>-Clinical data analysis<br>Ethical approval was given by the South Central-Oxford C Research Ethics Committee in England (reference 13/SC/0149), and by the Scotland A Research Ethics Committee (reference 20/SS/0028). The study was registered at https://www.isrctn.com/ISRCTN66726260	
Studies involving experimental animals: State details of authority granting ethics approval (IRB or equivalent committee(s), provide reference number for approval.		n/a

Studies involving specimen and field samples: State if relevant permits obtained, provide details of authority approving study; if none were required, explain why.		n/a

**Dual Use Research of Concern (DURC)**	**Yes (indicate where provided: page**	**n/a**
If study is subject to dual use research of concern, state the authority granting approval and reference number for the regulatory approval		n/a

Analysis

Attrition	Yes (indicate where provided: page no/section/legend)	n/a
State if sample or data point from the analysis is excluded, and whether the criteria for exclusion were determined and specified in advance.	Main text- Prenylated OAS1 is associated with less severe COVID-19<br>When adjusting for age, sex, and ethnicity, 24 cases were excluded as ethnicity was unknown and 6 cases excluded as age was not recorded. This was not specified in advance. "(unadjusted Odds Ratio (OR)=1.57, 95% CI 1.09, 2.25; following adjustment for age, sex, and ethnicity and exclusion of 30 cases with missing data OR=1.58, 95% CI 1.08, 2.30) (Fig. 5G)"	

Statistics	Yes (indicate where provided: page no/section/legend)	n/a
Describe statistical tests used and justify choice of tests.	Materials and methods - Clinical data analysis<br>Figure 5 legend	

Data Availability	Yes (indicate where provided: page no/section/legend)	n/a
State whether newly created datasets are available, including protocols for access or restriction on access.		n/a
If data are publicly available, provide accession number in repository or DOI or URL.	Materials and methods<br>https://doi.org/10.5525/gla.researchdata.1178<br>https://github.com/spyros-lytras/bat_OAS1<br>Raw iCLIP sequencing data are available from GEO under accession number GSE182394<br>isaric4c.net/data	
If publicly available data are reused, provide accession number in repository or DOI or URL, where possible.	Fig 2 legend.<br>-The Interferome v2.01 database (interferome.org)<br>-The Covid19 host genetics initiative (https://www.covid19hg.org/)	

Code Availability	Yes (indicate where provided: page no/section/legend)	n/a
For all newly generated code and software essential for replicating the main findings of the study:		
State whether the code or software is available.	Genome screening, PDE analysis and synteny analysis	
If code is publicly available, provide accession number in repository, or DOI or URL.	Materials and methods<br>https://github.com/spyros-lytras/bat_OAS1	

Reporting

Adherence to community standards	Yes (indicate where provided: page no/section/legend)	n/a
MDAR framework recommends adoption of discipline-specific guidelines, established and endorsed through community initiatives. Journals have their own policy about requiring specific guidelines and recommendations to complement MDAR.		
State if relevant guidelines (eg., ICMJE, MIBBI, ARRIVE) have been followed, and whether a checklist (eg., CONSORT, PRISMA, ARRIVE) is provided with the manuscript.		n/a
